# Multicomponent Synthesis of 2-(2,4-Diamino-3-cyano-5*H*-chromeno[2,3-*b*]pyridin-5-yl)malonic Acids in DMSO

**DOI:** 10.3390/molecules26226839

**Published:** 2021-11-12

**Authors:** Yuliya E. Ryzhkova, Michail N. Elinson, Oleg I. Maslov, Artem N. Fakhrutdinov

**Affiliations:** N. D. Zelinsky Institute of Organic Chemistry, Russian Academy of Sciences, 119991 Moscow, Russia; julia4912@mail.ru (Y.E.R.); mo100@yandex.ru (O.I.M.); fart@ioc.ac.ru (A.N.F.)

**Keywords:** salicylaldehyde, malononitrile dimer, malonic acid, chromeno[2,3-*b*]pyridine, multicomponent reactions, dimethyl sulfoxide

## Abstract

Dimethyl sulfoxide is a widely used solvent in organic synthesis and in the pharmaceutical industry because of its low cost, stability, and low toxicity. Multicomponent reactions are an advanced approach that has become an efficient, economical, and eco-friendly substitute for the conventional sequential multi-step synthesis of various biologically active compounds. This approach was adopted for the synthesis of previously unknown 2-(2,4-diamino-3-cyano-5*H*-chromeno[2,3-*b*]pyridin-5-yl)malonic acids via transformation of salicylaldehydes, malononitrile dimer, and malonic acid. It was shown that the use of DMSO at room temperature makes it possible to synthesize previously unavailable compounds. The investigation of the reaction mechanism using ^1^H-NMR monitoring made it possible to confirm the proposed mechanism of the transformation. The structure of synthesized 5*H*-chromeno[2,3-*b*]pyridines was confirmed by 2D-NMR spectroscopy.

## 1. Introduction

Multicomponent reactions (MCRs) are an important methodological arsenal in synthetic and medicinal chemistry [1]. A large number of publications that have appeared in this area over the past 5 years can confirm the significance of MCRs. This advanced approach has emerged as an efficient, economical, and eco-friendly substitute for the conventional sequential multi-step synthesis of various biologically active compounds [2]. MCRs exhibit a very high bond-forming index (BFI) as several non-hydrogen atom bonds are formed in one synthetic transformation [3]. Therefore, MCRs are the best strategy for the synthesis of complex heterocyclic structures.

Chromeno[2,3-*b*]pyridines are one of the important classes of condensed heterocyclic compounds from the point of view of medicinal chemistry. Depending on the substituents, they can exhibit different types of biological activity, such as antimicrobial [4], anticancer [5], antirheumatic [6], antimyopic [7], neuroprotective [8], and hypotensive [9] activities. Thus, the synthesis of a new type of chromeno[2,3-*b*]pyridines is an important goal for researchers.

Dimethyl sulfoxide (DMSO) is widely used as a solvent in organic synthesis and in the pharmaceutical industry because of its low cost, stability, and low toxicity [10]. Some of the characteristics of this polar solvent, such as its ability to stabilize charged intermediates and its high boiling point, are similar to those of dimethylacetamide, dimethylformamide (DMF), and *N*-methyl-2-pyrrolidone (NMP). However, DMSO is less toxic than other polar solvents and is extensively used as a solvent or an effective oxidant.

Not many types of MCRs have been carried out in DMSO, therefore, this is a promising area for research [11,12,13,14,15].

In the synthesis of chromeno[2,3-*b*]pyridines, both multistep classical and multicomponent methods [16] are applied. We have already published multicomponent syntheses of different types of chromeno[2,3-*b*]pyridines [17,18,19,20,21,22].

## 2. Results and Discussion

### 2.1. Multicomponent Synthesis of 2-(2,4-Diamino-3-cyano-5H-chromeno[2,3-b]pyridin-5-yl)malonic Acids ***4a**–**h***

Previously we reported the pot, atom, and step economy (PASE) synthesis of hydroxyquinolinone substituted chromeno[2,3-*b*]pyridines [21]. In this article, we presented ^1^H NMR real-time monitoring of the reaction in an NMR sample tube in DMSO-*d_6_* to confirm one of the proposed pathways of the transformation. The reaction in an NMR spectrometer proceeded efficiently and quickly. This fact gave us a reason to study the obtainment of chromeno[2,3-*b*]pyridines in DMSO already in a flask.

**Scheme 1:**
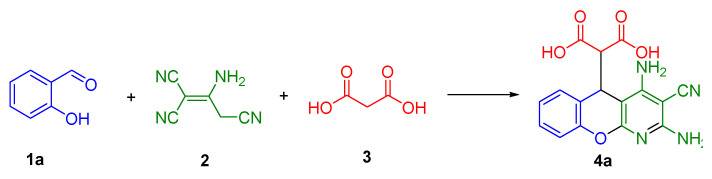
Reaction of salicylaldehyde **1a**, malononitrile dimer **2** and malonic acid **3**.

Initially, to examine the reaction of salicylaldehyde **1a**, malononitrile dimer **2**, and malonic acid **3**, we have carried out the multicomponent synthesis of chromeno[2,3-*b*]pyridine **4a** in high boiling point aprotic solvents (Scheme 1, Table 1, Entries 1–3).

When studying the reaction in DMSO, DMF, and NMP without heating for 24 h, the final compound **4a** was isolated in good yields of 70–92% (Table 1, Entries 1–3). For isolating **4a**, 15 mL of water was added to the reaction mixture.

Since the highest yield of chromeno[2,3-*b*]pyridine **4a** was achieved in DMSO, the reaction was further investigated in this solvent (Table 1, Entries 4–6). The reaction time (Table 1, Entry 4), the amount of water added to isolate compound **4a** (Table 1, Entry 5), and the reaction temperature (Table 1, Entry 6) were varied. However, in all these cases, it was not possible to increase the yield **4a**.

After that, we tried to carry out the transformation of the salicylaldehyde **1a**, malononitrile dimer **2**, and malonic acid **3** in our best previously found reaction systems (Table 1, Entries 7 and 8) [17,21]. However, refluxing the starting compounds in pyridine (Py) [17] did not result in compound **4a** formation (Table 1, Entry 7). When the reaction was carried out in an ethanol/pyridine mixture (3:1) [21], the yield of **4a** was only 15% (Table 1, Entry 8).

When the reaction in DMSO was finished, water was added to the reaction mixture and the final compound **4** was directly crystallized in pure form. Under the optimal conditions (Entry 1: stirring for 24 h in 5 mL of DMSO) multicomponent reactions of salicylaldehydes **1a**–**h**, malononitrile dimer **2**, and malonic acid **3** were carried out. 2-(2,4-Diamino-3-cyano-5*H*-chromeno[2,3-*b*]pyridin-5-yl)malonic acids **4a**–**h** were obtained in 65–98% yields (Table 2).

The substituent affects the yields of chromeno[2,3-*b*]pyridines **4**. Electron-donating methyl-, methoxy-, and ethoxy-groups decrease the yields of **4**. In the case of halogen substituents, the yield of compound **4** is increased. In the presence of both types of substituents (methoxy- and bromine, **4g**), the yield is average. This is due to the fact that halogens promote the delocalization of the negative charge, stabilize the intermediate anion, and thereby increase its acidity.

### 2.2. 2D-NMR Study of the Structure of Compound ***4f***

The structure of compound **4f** was confirmed by NMR spectroscopy.

**Figure 1:**
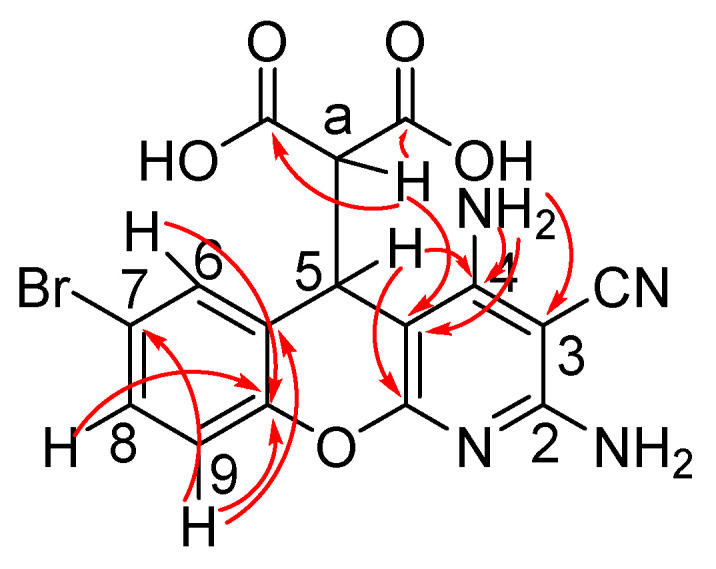
The structure of compound **4f**. Key ^1^H-^13^C-HMBC spectrum correlations established by NMR are shown by arrows.

The proton spectrum contained signals from all groups, including carboxyl fragments (broad signal at 12.8 ppm). The benzene fragment is substituted at position 7, as evidenced by the characteristic set of signals in the proton spectrum (two doublets at 7.65 and 7.01 ppm and one doublet of doublets at 7.43 ppm). The signals of the amino groups of the pyridine ring (*δ* 6.60 and 6.48 ppm) could be distinguished due to the detected correlation in the ^1^H-^13^C-HMBC spectrum of 4-NH_2_ with C^4a^ (*δ*_H/C_ 6.60/87.7 ppm) (Figure 1). The carbon signal of the third position has a very upfield chemical shift (*δ* 71.1 ppm). This chemical shift is due to the substitution of the nitrile group, and the shielding of the nucleus by electrons of the triple bond. In position 5, there is a malonic acid residue. This is confirmed by the spin interaction from the HMBC spectrum of H^a^ with the carbons of the benzene and pyridine rings (Figure 1).

Complete correlation of signals in ^1^H and ^13^C-NMR spectra of chromeno[2,3-*b*]pyridine **4f**:

^1^H-NMR (600 MHz, DMSO-*d*_6_) *δ*: 12.80 (br s, 2H, COOH), 7.65 (d, ^4^*J* = 2.5 Hz, 1H, H^6^), 7.43 (dd, ^3^*J* = 8.6 Hz, ^4^*J* = 2.5 Hz, 1H, H^8^), 7.01 (d, ^3^*J* = 8.7 Hz, 1H, H^9^), 6.60 (s, 2H, 4-NH_2_), 6.48 (s, 2H, 2-NH_2_), 4.80 (d, ^3^*J* = 4.8 Hz, 1H, H^5^), 3.44 (d, ^3^*J* = 4.8 Hz, 1H, H^a^) ppm.

^13^C-NMR (151 MHz, DMSO-*d*_6_) *δ*: 169.1 (COOH), 168.9 (COOH), 160.5 (C^4^), 159.7 (C^2^), 156.6 (C^1a^), 151.5 (C^9a^), 131.8 (C^6^), 131.0 (C^8^), 125.0 (C^6a^), 118.3 (C^9^), 116.4 (CN), 114.7 (C^7^), 87.7 (C^4a^), 71.1 (C^3^), 56.8 (C^a^), 32.5 (C^5^) ppm.

Two-dimensional (2D) NMR spectra of the compound **4f** are presented in Supplementary Materials (Figures S17 and S18).

### 2.3. ^1^H-NMR Reaction Monitoring

**Figure 2:**
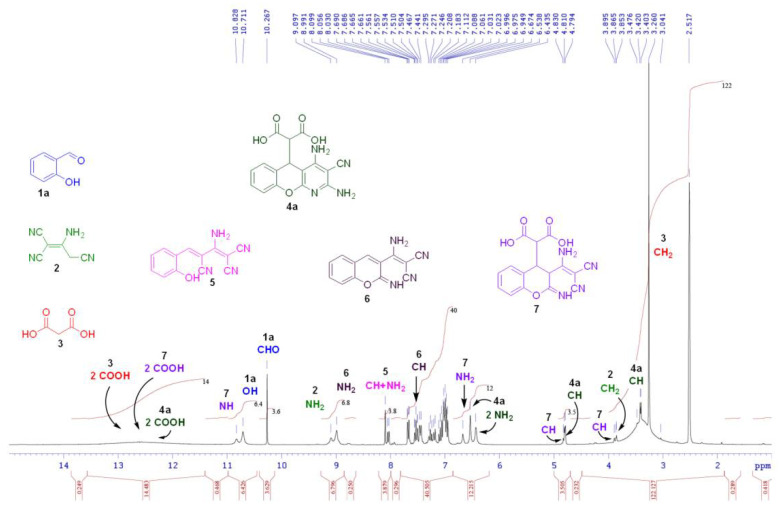
Representative ^1^H-NMR spectrum of multicomponent reaction in DMSO-*d_6_* at 313 K recorded 45 min after dissolution.

We assumed that the reaction proceeds according to the standard mechanism, which we proved earlier [21]. However, to prove our assumption, the reaction was monitored using ^1^H-NMR spectroscopy (Figure 2).

To reduce the influence of sample preparation, the transformation of starting materials into chromeno[2,3-*b*]pyridine **4a** was carried out and monitored directly in an NMR sample tube into a spectrometer without catalyst in DMSO-*d_6_* to slow down the reaction.

During the NMR study, seven major components were recorded: salicylaldehyde **1a**, malononitrile dimer **2**, malonic acid **3**, intermediate **5**, intermediate **6**, intermediate **7**, and chromeno[2,3-*b*]pyridine **4a**. A representative ^1^H-NMR spectrum with the assignment of peaks showed in Figure 2. NMR spectra of the monitoring are presented in Supplementary Materials (Figure S19–S23).

As shown in Figure 2, malononitrile dimer **2** is consumed quickly, and the Knoevenagel adduct **5** is formed. Compound **5** cyclizes to intermediate **6**. In this spectrum, we also found intermediate **7**, which is the final compound of the Michael reaction. Also in Figure 2, the target chromeno[2,3-*b*]pyridine **4a** is recorded.

**Scheme 2:**
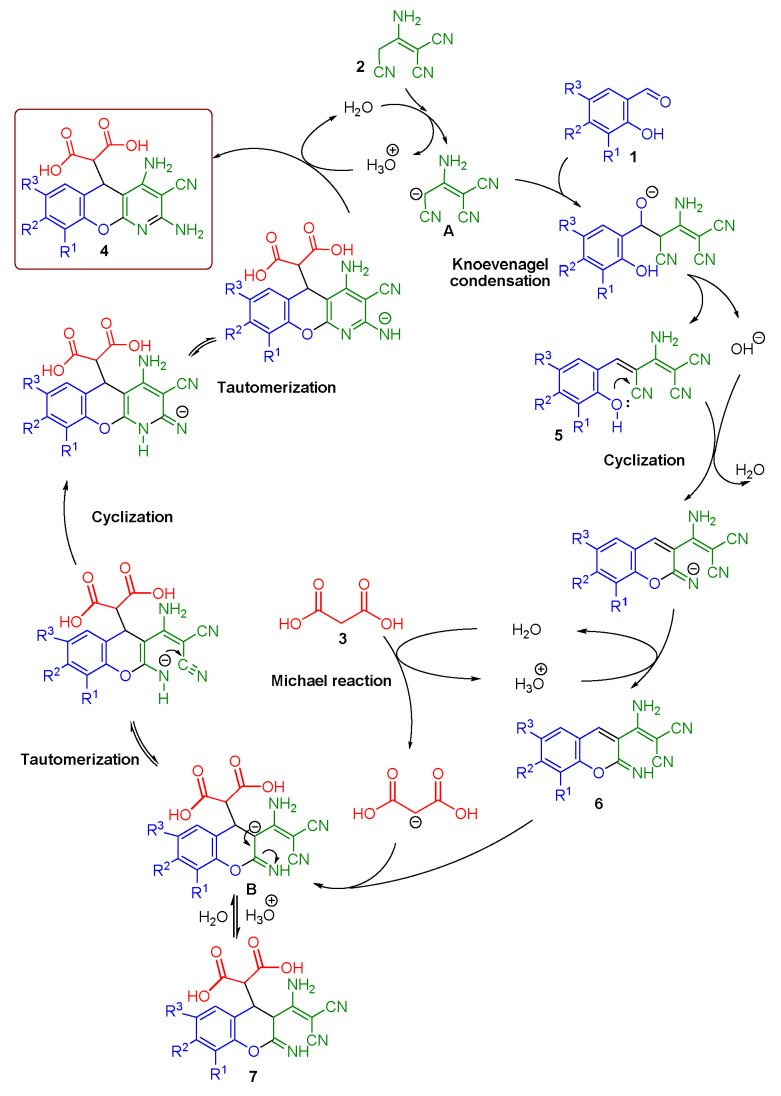
Mechanism of salicylaldehydes **1**, malononitrile dimer **2**, and malonic acid **3** transformation into chromeno[2,3-*b*]pyridines **4**. Catalytic cycles are simplified.

Based on the above data and taking into consideration earlier published results [19,21], we suggest that the first stage was a rapid formation of intermediate **5** with the expulsion of a hydroxide anion [23] (Scheme 2). This hydroxide anion instantly catalyzed a rapid cyclization of intermediate **5** into intermediate **6**. Then, the Michael addition of malonic acid **3** occurs to form anion **B** (as well as intermediate **7**). Next, there are successive cyclizations and isomerization to the final chromeno[2,3-*b*]pyridine **4**.

## 3. Materials and Methods

### 3.1. General Information

The solvents and reagents were purchased from commercial sources and used as received. 2-Aminoprop-1-ene-1,1,3-tricarbonitrile **2** was obtained from malononitrile according to the literature [24].

All melting points were measured with a Gallenkamp melting-point apparatus (Gallenkamp & Co., Ltd, London, UK) and were uncorrected. ^1^H and ^13^C-NMR spectra were recorded in DMSO-*d_6_* with Bruker AM300, Bruker AV500, and Bruker AV600 spectrometers (Bruker Corporation, Billerica, MA, USA) at ambient temperature. Chemical shift values are relative to Me_4_Si. Two-dimensional (2D) NMR spectra were registered with a Bruker AV500 spectrometer. 1H NMR monitoring spectra were registered with a Bruker AM300 spectrometer (Bruker Corporation, Billerica, MA, USA). The IR spectrum was recorded with a Bruker ALPHA-T FT-IR spectrometer (Bruker Corporation, Billerica, MA, USA) in a KBr pellet. MS spectra (EI = 70 eV) were obtained directly with a Kratos MS-30 spectrometer (Kratos Analytical Ltd, Manchester, UK). High-resolution mass spectra (HRMS) were measured on a Bruker micrOTOF II (Bruker Corporation, Billerica, MA, USA) instrument using electrospray ionization (ESI).

### 3.2. Synthesis of 2-(2,4-Diamino-3-cyano-5H-chromeno[2,3-b]pyridin-5-yl)malonic Acids ***4a**–**h***

Salicylaldehyde 1a–h (1 mmol), 2-aminoprop-1-ene-1,1,3-tricarbonitrile 2 (0.132 g, 1 mmol) and malonic acid 3 (0.104 g, 1 mmol) were stirred in 5 mL of DMSO for 24 h at ambient temperature. After the reaction was completed, 15 mL of water was added to the solution. The formed solid was filtered, washed with well-chilled ethanol (3 mL × 2 mL), and dried to isolate pure substituted 2-(2,4-diamino-3-cyano-5*H*-chromeno[2,3-*b*]pyridin-5-yl)malonic acids **4a**–**h**.

2-(2,4-Diamino-3-cyano-5*H*-chromeno[2,3-*b*]pyridin-5-yl)malonic acid **4a**, (yellowish powder, 0.313 g, 92%), mp 214–215 °C (decomp.) (from DMSO-H_2_O), FTIR (KBr) cm^−1^: 3412, 3323, 3230, 3083, 2223, 1718, 1654, 1461, 1329, 1267. ^1^H-NMR (300 MHz, DMSO-*d_6_*) *δ* 3.41 (d, ^3^*J* = 5.2 Hz, 1H, malonic), 4.79 (d, ^3^*J* = 5.2 Hz, 1H, CH(5)), 6.44 (br s, 2H, NH_2_), 6.53 (s, 2H, NH_2_), 7.12–6.99 (m, 2H, 2 CH Ar), 7.26 (t, ^3^*J* = 7.5 Hz, 1H, CH Ar), 7.45 (d, ^3^*J* = 7.5 Hz, 1H, CH Ar), 13.22–12.15 (br s, 2H, 2 COOH) ppm. ^13^C-NMR (75 MHz, DMSO-*d_6_*) *δ* 33.3, 58.0, 71.4, 88.9, 116.5, 117.0, 123.3, 123.9, 128.7, 129.7, 152.5, 157.1, 160.1, 161.4, 169.5, 169.6 ppm. MS (EI, 70 eV) *m*/*z* (%): 296 ([M-CO_2_]^+^, 1), 277 (12), 248 (3), 237 (100), 209 (4), 171 (17), 145 (2), 78 (5), 44 (26), 18 (24). HRMS-ESI: *m/z* [M + H]^+^, calcd for C_16_H_13_N_4_O_5_ 341.0880, found 341.0878.

2-(2,4-Diamino-3-cyano-8-methoxy-5*H*-chromeno[2,3-*b*]pyridin-5-yl)malonic acid **4b**, (yellowish powder, 0.244 g, 66%), mp 225–226 °C (decomp.). (from DMSO-H_2_O), FTIR (KBr) cm^−1^: 3474, 3358, 3252, 3104, 2213, 1722, 1650, 1480, 1334, 1208. ^1^H-NMR (300 MHz, DMSO-*d_6_*) *δ* 3.39 (d, ^3^*J* = 4.6 Hz, 1H, malonic), 3.74 (s, 3H, OMe), 4.71 (d, ^3^*J* = 4.6 Hz, 1H, CH(5)), 6.44 (br s, 2H, NH_2_), 6.52 (s, 2H, NH_2_), 6.61 (s, 1H, CH Ar), 6.66 (d, ^3^*J* = 8.6 Hz, 1H, CH Ar), 7.36 (d, ^3^*J* = 8.6 Hz, 1H, CH Ar), 13.21–12.21 (br s, 2H, 2 COOH) ppm. ^13^C-NMR (75 MHz, DMSO-*d_6_*) *δ* 32.3, 55.3, 57.4, 70.9, 88.9, 101.3, 109.7, 114.6, 116.5, 129.8, 152.8, 156.5, 159.2, 159.5, 160.8, 169.1, 169.3 ppm. MS (EI, 70 eV) *m*/*z* (%): 307 ([M-CO2-H2O-H]^+^, 4), 280 (9), 267 (100), 224 (19), 195 (4), 171 (2), 134 (2), 77 (3), 44 (32), 18 (55). HRMS-ESI: *m/z* [M + H]^+^, calcd for C_17_H_15_N_4_O_6_ 371.0992, found 371.0988.

2-(2,4-Diamino-3-cyano-9-ethoxy-5*H*-chromeno[2,3-*b*]pyridin-5-yl)malonic acid **4c**, (yellowish powder, 0.307g, 80%), mp 191–192 °C (decomp.) (from DMSO-H_2_O), FTIR (KBr) cm^−1^: 3447, 3380, 3064, 2985, 2216, 1729, 1664, 1469, 1392, 1281. ^1^H-NMR (300 MHz, DMSO-*d_6_*) *δ* 1.37 (t, ^3^*J* = 6.9 Hz, 3H, CH_3_), 3.39 (d, ^3^*J* = 5.3 Hz, 1H, malonic), 4.06 (q, ^3^*J* = 6.9 Hz, 2H, OCH_2_), 4.78 (d, ^3^*J* = 5.3 Hz, 1H, CH(5)), 6.52 (br s, 4H, 2 NH_2_), 7.05–6.89 (m, 3H, 3 CH Ar), 13.28–12.11 (br s, 2H, 2 COOH) ppm. ^13^C-NMR (75 MHz, DMSO-*d_6_*) *δ* 14.8, 33.0, 57.9, 63.9, 70.9, 88.5, 112.1, 116.6, 120.4, 123.2, 123.9, 141.4, 146.5, 156.7, 159.6, 161.0, 169.1 (2C) ppm. MS (EI, 70 eV) *m*/*z* (%): 340 ([M-CO_2_]^+^, 1), 320 (8), 281 (100), 253 (67), 237 (18), 187 (10), 170 (4), 92 (4), 60 (15), 29 (90). HRMS-ESI: *m/z* [M + H]^+^, calcd for C_18_H_17_N_4_O_6_ 385.1148, found 385.1150.

2-(2,4-Diamino-3-cyano-7-methyl-5*H*-chromeno[2,3-*b*]pyridin-5-yl)malonic acid **4d**, (yellowish powder, 0.230 g, 65%), mp 186–187 °C (decomp.) (from DMSO-H_2_O), FTIR (KBr) cm^−1^: 3446, 3367, 3193, 3105, 2216, 1722, 1665, 1449, 1404, 1287. ^1^H-NMR (300 MHz, DMSO-*d_6_*) *δ* 2.26 (s, 3H, CH_3_), 3.41 (d, ^3^*J* = 4.8 Hz, 1H, malonic), 4.74 (d, ^3^*J* = 4.8 Hz, 1H, CH(5)), 6.42 (br s, 2H, NH_2_), 6.51 (s, 2H, NH_2_), 6.94 (d, ^3^*J* = 7.7 Hz, 1H, CH Ar), 7.07 (d, ^3^*J* = 7.7 Hz, 1H, CH Ar), 7.24 (s, 1H, CH Ar), 13.55–11.77 (br s, 2H, 2 COOH) ppm. ^13^C-NMR (75 MHz, DMSO-*d_6_*) *δ* 20.5, 32.9, 57.7, 70.9, 88.5, 115.8, 116.6, 122.7, 128.7, 129.4, 132.3, 149.9, 156.7, 159.6, 161.1, 169.2 (2C) ppm. MS (EI, 70 eV) *m*/*z* (%): 291 ([M-CO_2_-H_2_O-H]^+^, 33), 262 (11), 251 (100), 237 (8), 185 (16), 164 (1), 140 (5), 125 (8), 77 (11), 44 (50). HRMS-ESI: *m/z* [M + H]^+^, calcd for C_17_H_15_N_4_O_5_ 355.1042, found 355.1038.

2-(2,4-Diamino-7-chloro-3-cyano-5*H*-chromeno[2,3-*b*]pyridin-5-yl)malonic acid **4e**, (yellowish powder, 0.367 g, 98%), mp 193–194 °C (decomp.) (from DMSO-H_2_O), FTIR (KBr) cm^−1^: 3341, 3223, 3153, 3083, 2217, 1727, 1655, 1484, 1402, 1229. ^1^H-NMR (300 MHz, DMSO-*d_6_*) *δ* 3.47 (d, ^3^*J* = 4.6 Hz, 1H, malonic), 4.82 (d, ^3^*J* = 4.6 Hz, 1H, CH(5)), 6.52 (br s, 2H, NH_2_), 6.63 (s, 2H, NH_2_), 7.09 (d, ^3^*J* = 8.7 Hz, 1H, CH Ar), 7.33 (dd, ^3^*J* = 8.7 Hz, ^4^*J* = 2.2 Hz, 1H, CH Ar), 7.54 (d, ^4^*J* = 2.2 Hz, 1H, CH Ar), 13.55–12.09 (br s, 2H, 2 COOH) ppm. ^13^C-NMR (75 MHz, DMSO-*d_6_*) *δ* 32.6, 56.9, 71.1, 87.7, 116.5, 117.9, 124.5, 126.8, 128.2, 129.0, 151.0, 156.6, 159.7, 160.5, 168.9, 169.1 ppm. MS (EI, 70 eV) *m*/*z* (%): 313 (^37^Cl, [M-CO_2_-H_2_O]^+^, 24), 311 (^35^Cl, [M-CO_2_-H_2_O]^+^, 63), 273 (^37^Cl, 32), 271 (^35^Cl, 100), 243 (9), 205 (22), 179 (3), 152 (5), 114 (6), 89 (7), 66 (46), 42 (82). HRMS-ESI: *m/z* [M + H]^+^, calcd for C_16_H_12_ClN_4_O_5_ 377.0467 (^37^Cl), 375.0496 (^35^Cl), found 377.0463 (^37^Cl), 375.0493 (^35^Cl).

2-(2,4-Diamino-7-bromo-3-cyano-5*H*-chromeno[2,3-*b*]pyridin-5-yl)malonic acid **4f**, (white powder, 0.398 g, 95%), mp 165–166 °C (decomp.) (from DMSO-H_2_O), FTIR (KBr) cm^−1^: 3365, 3222, 2223, 1722, 1657, 1561, 1480, 1341, 1265, 1226. ^1^H-NMR (300 MHz, DMSO-*d_6_*) *δ* 3.46 (d, ^3^*J* = 4.6 Hz, 1H, malonic), 4.82 (d, ^3^*J* = 4.6 Hz, 1H, CH(5)), 6.49 (br s, 2H, NH_2_), 6.61 (s, 2H, NH_2_), 7.03 (d, ^3^*J* = 8.6 Hz, 1H, CH Ar), 7.45 (dd, ^3^*J* = 8.6 Hz, ^4^*J* = 2.1 Hz, 1H, CH Ar), 7.67 (d, ^4^*J* = 2.1 Hz, 1H, CH Ar), 13.57–12.10 (br s, 2H, 2 COOH) ppm. ^13^C-NMR (DMSO-*d_6_*, 75 MHz) *δ* 32.5, 56.8, 71.1, 87.7, 114.7, 116.4, 118.3, 125.0, 131.0, 131.8, 151.5, 156.6, 159.6, 160.5, 168.9, 169.1 ppm. MS (EI, 70 eV) *m*/*z* (%): 357 (^81^Br, [M-CO_2_-H_2_O]^+^, 65), 355 (^79^Br, [M-CO_2_-H_2_O]^+^, 65), 316 (21), 277 (21), 249 (9), 220 (3), 165 (7), 124 (8), 88 (9), 66 (41), 42 (100). HRMS-ESI: *m/z* [M + H]^+^, calcd for C_16_H_12_BrN_4_O_5_ 420.9971 (^81^Br), 418.9991 (^79^Br), found 420.9974 (^81^Br), 418.9993 (^79^Br).

2-(2,4-Diamino-7-bromo-3-cyano-9-methoxy-5*H*-chromeno[2,3-*b*]pyridin-5-yl)malonic acid **4g**, (white powder, 0.355 g, 79%), mp 228–229 °C (decomp.) (from DMSO-H_2_O), FTIR (KBr) cm^−1^: 3436, 3345, 3087, 2214, 1727, 1662, 1580, 1489, 1396, 1231. ^1^H-NMR (300 MHz, DMSO-*d_6_*) *δ* 3.42 (d, ^3^*J* = 4.8 Hz, 1H, malonic), 4.79 (d, ^3^*J* = 4.8 Hz, 1H, CH(5)), 6.48 (br s, 2H, NH_2_), 6.59 (s, 2H, NH_2_), 7.15 (s, 1H, CH Ar), 7.23 (s, 1H, CH Ar), 13.41–12.20 (br s, 2H, 2 COOH) ppm. ^13^C-NMR (DMSO-*d_6_*, 126 MHz) *δ* 169.1, 168.9, 160.6, 159.7, 156.7, 148.3, 141.0, 125.3, 123.1, 116.5, 114.5, 114.2, 87.9, 71.1, 57.2, 56.2, 32.7 ppm. MS (EI, 70 eV) *m*/*z* (%): 387 (^81^Br, [M-CO_2_-H_2_O]^+^, 13), 385 (^79^Br, [M-CO_2_-H_2_O]^+^, 14), 347 (^81^Br, 15), 345 (^79^Br, 15), 207 (2), 264 (1), 235 (2), 195 (4), 100 (2), 66 (17), 42 (34), 15 (100). HRMS-ESI: *m/z* [M + H]^+^, calcd for C_17_H_14_BrN_4_O_6_ 451.0076 (^81^Br), 449.0097 (^79^Br), found 451.0072 (^81^Br), 449.0094 (^79^Br).

2-(9,11-Diamino-10-cyano-12*H*-benzo[5,6]chromeno[2,3-*b*]pyridin-12-yl)malonic acid **4h**, (beige powder, 0.254 g, 65%), mp 220–221 °C (decomp.) (from DMSO-H_2_O), FTIR (KBr) cm^−1^: 3428, 3395, 3025, 2220, 1731, 1666, 1598, 1449, 1294, 1237. ^1^H-NMR (300 MHz, DMSO-*d_6_*) *δ* 3.37 (d, ^3^*J* = 2.9 Hz, 1H, malonic), 5.41 (d, ^3^*J* = 2.9 Hz, 1H, CH(5)), 6.52 (br s, 2H, NH_2_), 6.87 (s, 2H, NH_2_), 7.34 (d, ^3^*J* = 8.9 Hz, 1H, CH Ar), 7.51 (t, ^3^*J* = 7.2 Hz, 1H, CH Ar), 7.64 (t, ^3^*J* = 7.2 Hz, 1H, CH Ar), 8.04–7.85 (m, 3H, 3 CH Ar), 13.69–12.20 (br s, 2H, 2 COOH), ppm. ^13^C-NMR (75 MHz, DMSO-*d_6_*) *δ* 29.5, 59.7, 70.9, 87.9, 116.7, 116.9, 117.4, 122.3, 124.7, 127.4, 128.9, 129.2, 129.9, 130.7, 149.3, 157.6, 159.7, 160.8, 168.9, 171.5 ppm. MS (EI, 70 eV) *m*/*z* (%): 340 ([M-CO_2_-H_2_O-H]^+^, 6), 287 (100), 237 (2), 221 (11), 177 (3), 144 (5), 139 (5), 92 (2), 66 (22), 44 (19). HRMS-ESI: *m/z* [M + H]^+^, calcd for C_20_H_15_N_4_O_5_ 391.1042, found 391.1040.

## 4. Conclusions

In summary, the PASE transformation of salicylaldehydes, malononitrile dimer, and malonic acid into previously unknown 2-(2,4-diamino-3-cyano-5*H*-chromeno[2,3-*b*]pyridin-5-yl)malonic acids has been found. The developed DMSO-based approach is facile and easy for isolating final compounds directly from the reaction mixture using water addition and the yields of final compounds are 65–98%. This reaction is the first example of a multicomponent synthesis of chromeno[2,3-*b*]pyrnidines in DMSO.

During the investigation of the reaction mechanism using ^1^H-NMR monitoring, it was determined that the multicomponent process proceeds according to the usual mechanism confirmed by us earlier. Two-dimensional (2D) NMR spectroscopy confirmed the proposed structure of synthesized 5*H*-chromeno[2,3-*b*]pyridines.

## Figures and Tables

**Table 1:** Multicomponent synthesis of 2-(2,4-diamino-3-cyano-5*H*-chromeno[2,3-*b*]pyridine-5-yl)malonic acid **4a** ^1^.

Entry	Solvent	Time (h)	Temperature (°C)	Water Volume (mL)	Yield (%)
1	DMSO	24	23 (rt)	15	92 ^2^
2	DMF	24	23 (rt)	15	75 ^2^
3	NMP	24	23 (rt)	15	70 ^2^
4	DMSO	12	23 (rt)	15	62 ^2^
5	DMSO	24	23 (rt)	10	81 ^2^
6	DMSO	24	40	15	66
7	Py	4	115	-	-
8	EtOH/Py (3:1)	4	81	-	15

^1^ Reaction conditions: salicylaldehyde **1a** (1 mmol), malononitrile dimer **2** (1 mmol), malonic acid **3** (1 mmol) were stirred in 5 mL of solvent, then H_2_O was added. ^2^ Isolated yield, in other cases NMR data.

**Table 2:** Multicomponent reaction of salicylaldehydes **1a**–**h**, malononitrile dimer **2** and malonic acid **3** ^1^.

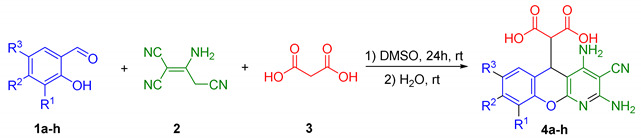
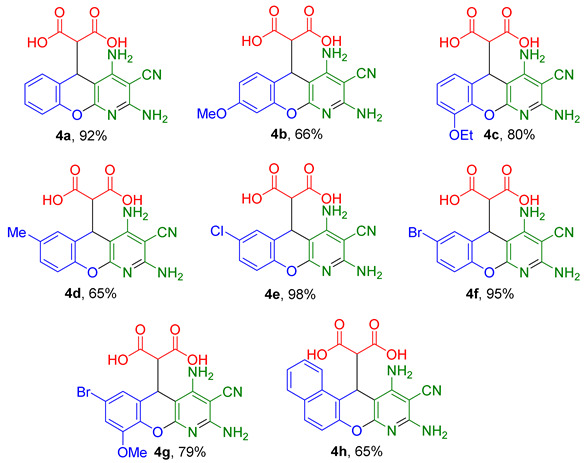

^1^ Reaction conditions: salicylaldehyde **1a**–**h** (1 mmol), malononitrile dimer **2** (1 mmol), malonic acid **3** (1 mmol) were stirred in 5 mL of DMSO for 24 h, then 15 mL of H_2_O was added. Isolated yields.

## Data Availability

Data is contained within the article or supplementary material.

## Supplementary Materials

The following are available online, Figures S1–S16: ^1^H and ^13^C Spectra of synthesized compounds **4a**–**h**, Figures S17–S18: 2D-NMR Spectra of **4f**, Figures S19-S23: ^1^H-NMR monitoring spectra (300 MHz, 313 K).

## Sample Availability

Samples of the compounds **2a**–**h** are available from the authors.
