# Supplementary material for: Multicomponent Synthesis of 2-(2,4-Diamino-3-cyano-5H-chromeno[2,3-b]pyridin-5-yl)malonic Acids in DMSO
Source: Molecules. 2021 Nov 12;26(22):6839. doi: 10.3390/molecules26226839 (PMC8624304; doi:10.3390/molecules26226839)

Supplementary Material:

Multicomponent Synthesis of 2-(2,4-Diamino-3-cyano-5*H*-chromeno[2,3-*b*]pyridin-5-yl)malonic Acids in DMSO

Yuliya E. Ryzhkova*, Oleg I. Maslov, Artem N. Fakhrutdinov, Michail N. Elinson

N. D. Zelinsky Institute of Organic Chemistry, Russian Academy of Sciences, 47 Leninsky Pr., Moscow 119991, Russian Federation. E-mail: elinson@ioc.ac.ru

Table of Contents

1. The copies of ^{1}H NMR and ^{13}C NMR spectra for compounds **4a-h**..S2

2. 2D NMR data for compound **4f**..S10

3. ^{1}H NMR monitoring spectra (300 MHz, 313 K)..S11

1. The copies of ^{1}H NMR and ^{13}C NMR spectra for compounds 4a-h

Figure S1. ^{1}H NMR spectrum of 2-(2,4-diamino-3-cyano-5*H*-chromeno[2,3-*b*]pyridin-5-yl)malonic acid **4a** in DMSO-d_6.

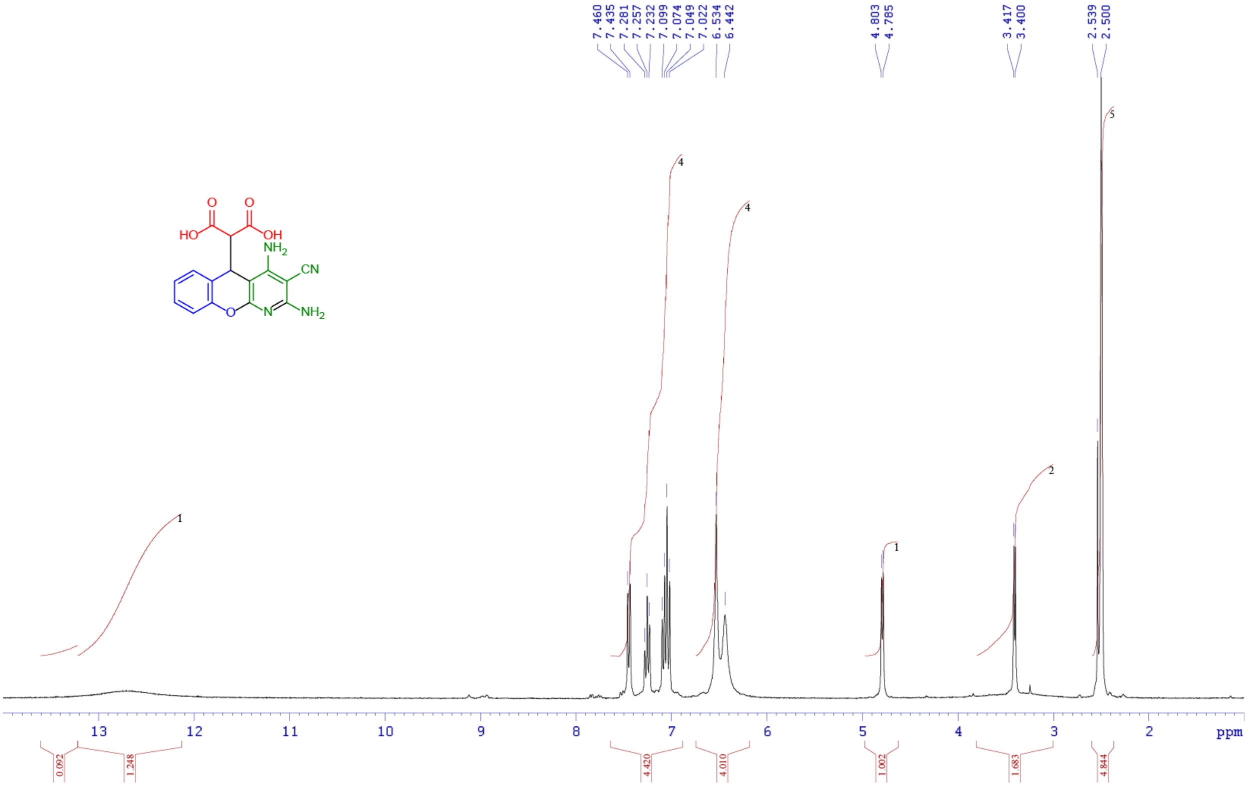

Figure S2. ^{13}C NMR spectrum of 2-(2,4-diamino-3-cyano-5*H*-chromeno[2,3-*b*]pyridin-5-yl)malonic acid **4a** in DMSO-d_6.

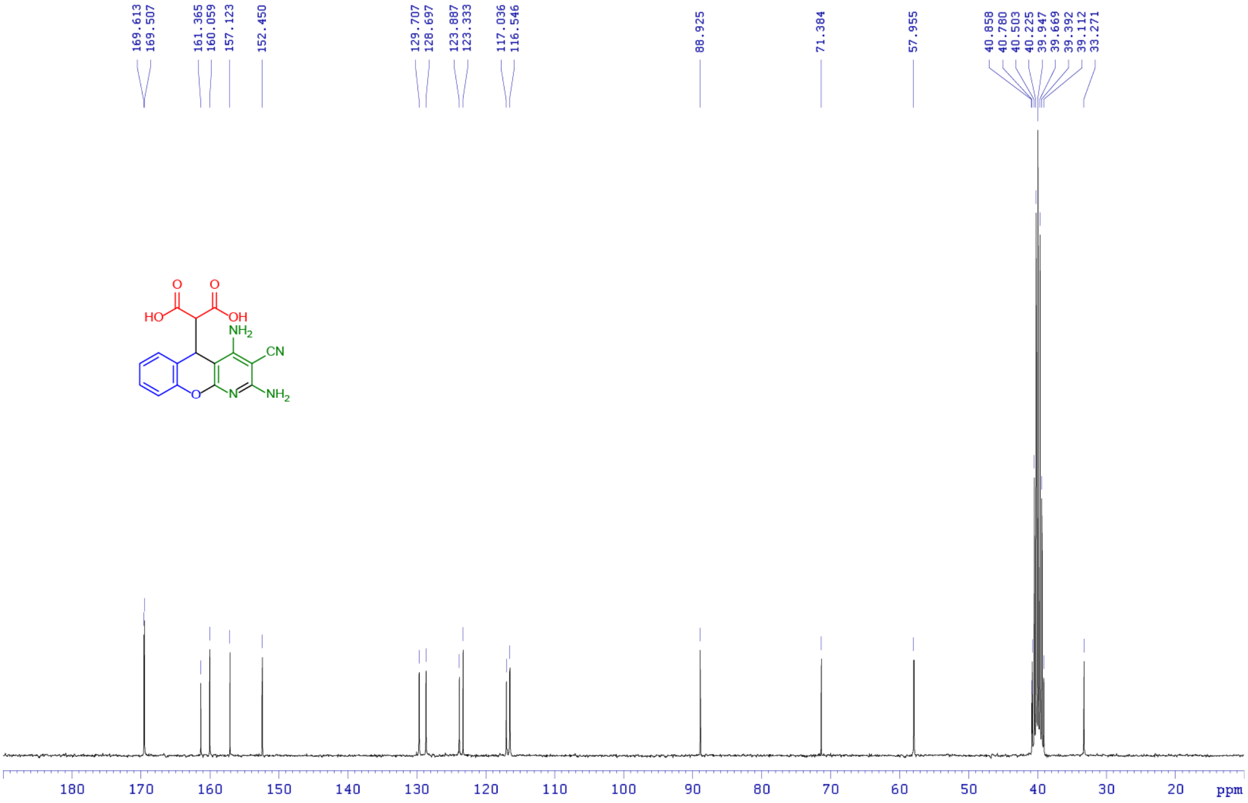

Figure S3. ^{1}H NMR spectrum of 2-(2,4-diamino-3-cyano-8-methoxy-5*H*-chromeno[2,3-*b*]-pyridin-5-yl)malonic acid **4b** in DMSO-*d*6.

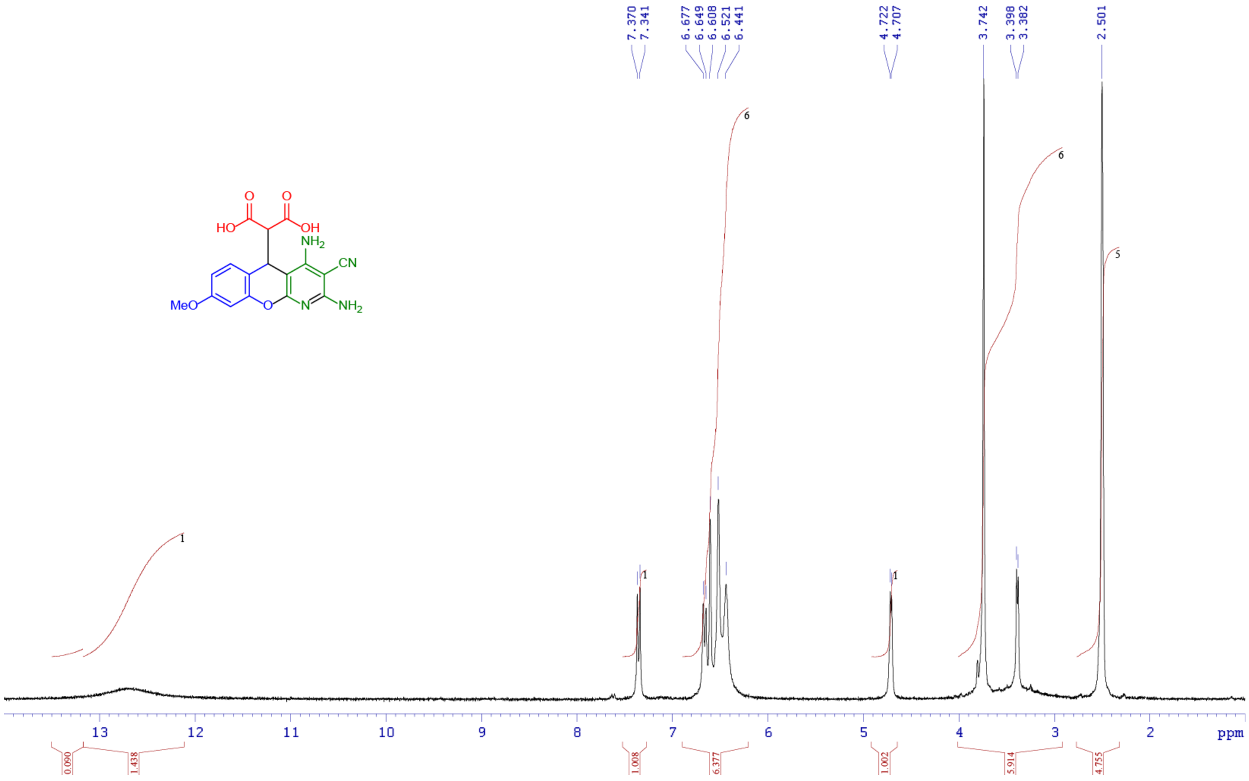

Figure S4. ^{13}C NMR spectrum of 2-(2,4-diamino-3-cyano-8-methoxy-5*H*-chromeno[2,3-*b*]-pyridin-5-yl)malonic acid **4b** in DMSO-*d*6.

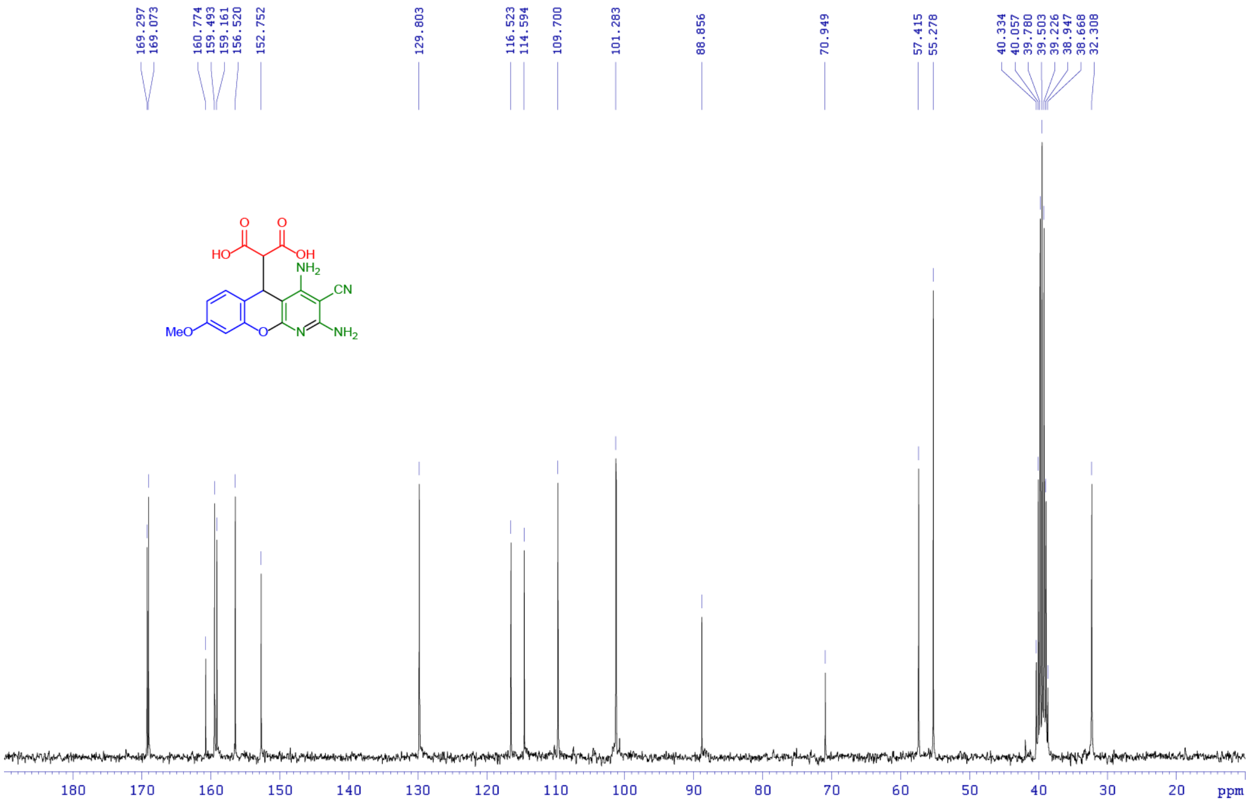

Figure S5. ^{1}H NMR spectrum of 2-(2,4-diamino-3-cyano-9-ethoxy-5*H*-chromeno[2,3-*b*]pyridin-5-yl)malonic acid **4c** in DMSO-d_6.

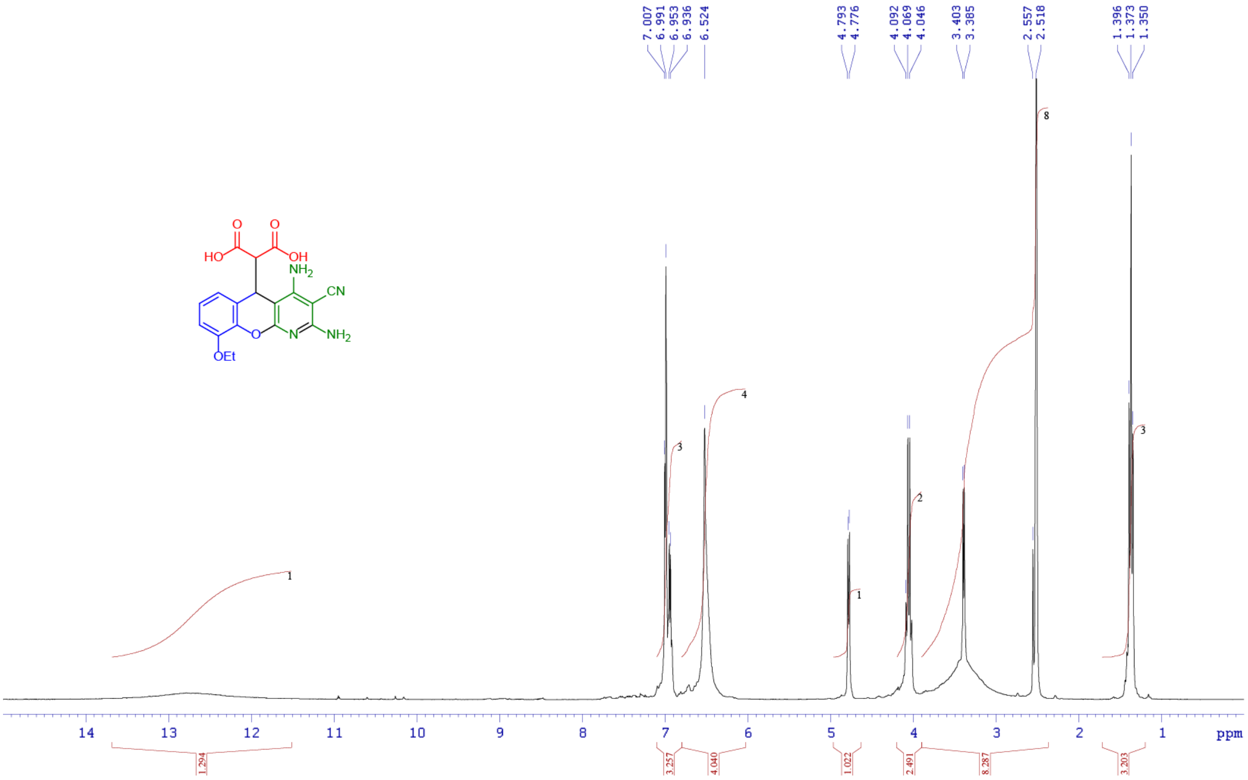

Figure S6. ^{13}C NMR spectrum of 2-(2,4-diamino-3-cyano-9-ethoxy-5*H*-chromeno[2,3-*b*]pyridin-5-yl)malonic acid **4c** in DMSO-d_6.

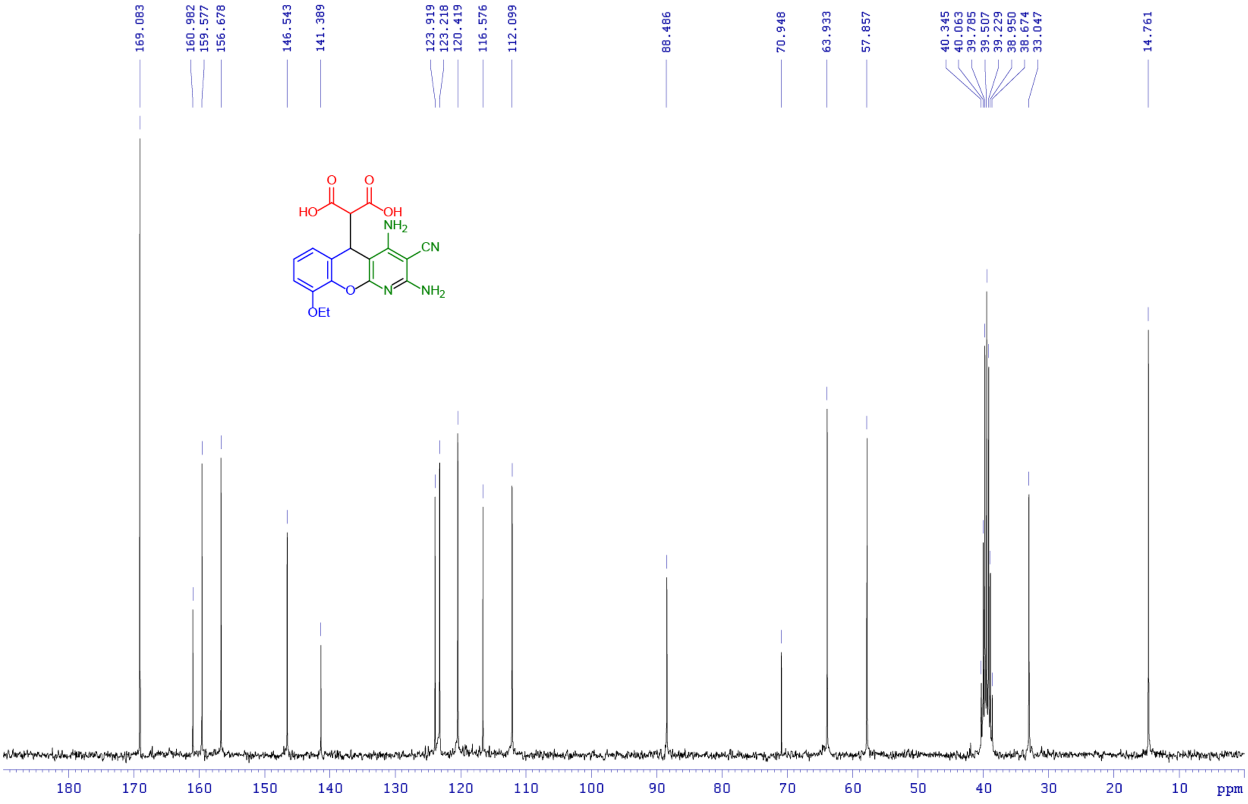

Figure S7. ^{1}H NMR spectrum of 2-(2,4-diamino-3-cyano-7-methyl-5*H*-chromeno[2,3-*b*]pyridin-5-yl)malonic acid **4d** in DMSO-d_6.

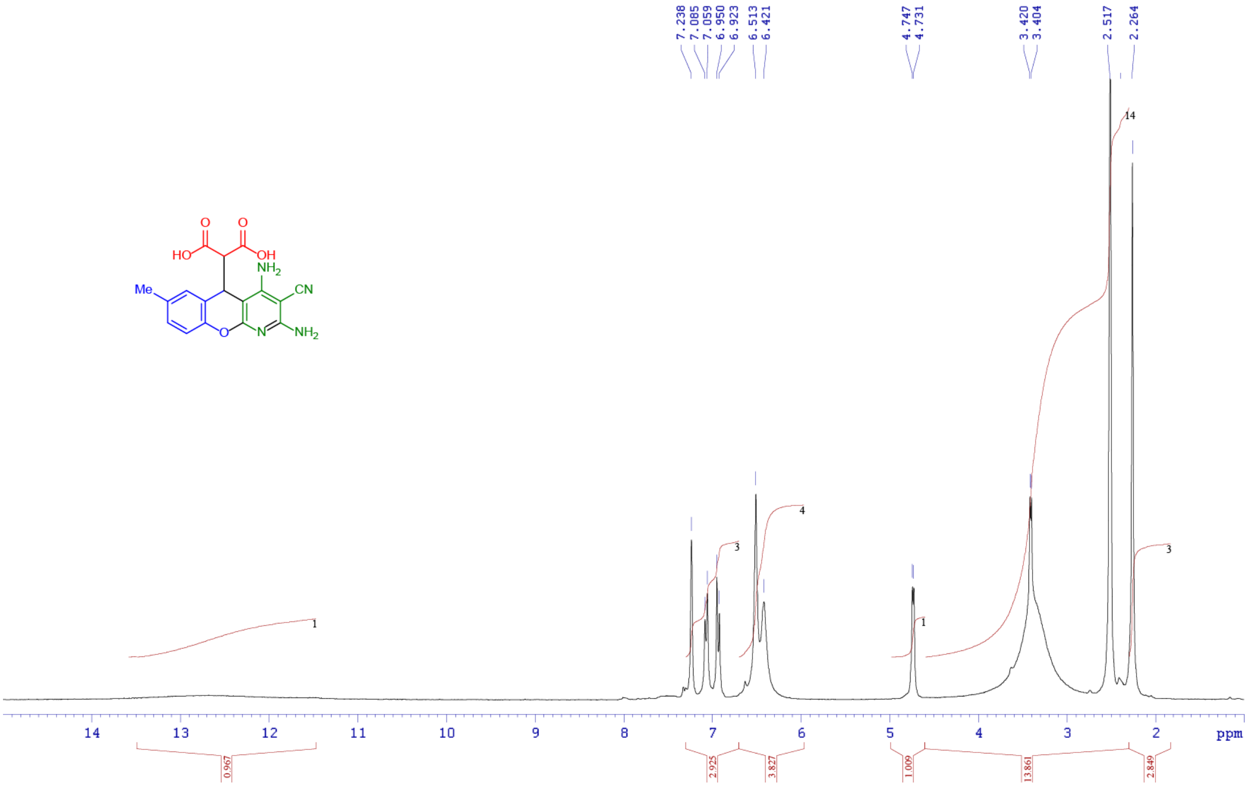

Figure S8. ^{13}C NMR spectrum of 2-(2,4-diamino-3-cyano-7-methyl-5*H*-chromeno[2,3-*b*]pyridin-5-yl)malonic acid **4d** in DMSO-d_6.

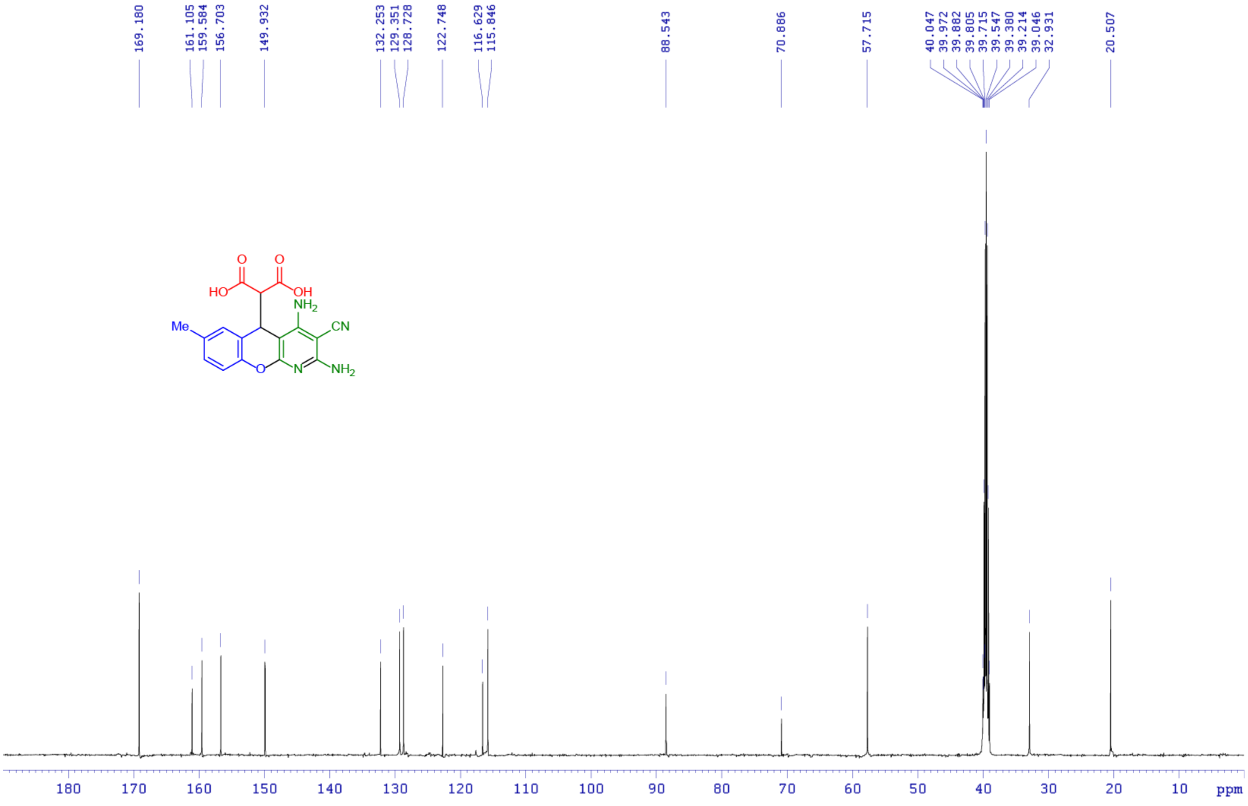

Figure S9. ^{1}H NMR spectrum of 2-(2,4-diamino-7-chloro-3-cyano-5*H*-chromeno[2,3-*b*]pyridin-5-yl)malonic acid **4e** in DMSO-d_6.

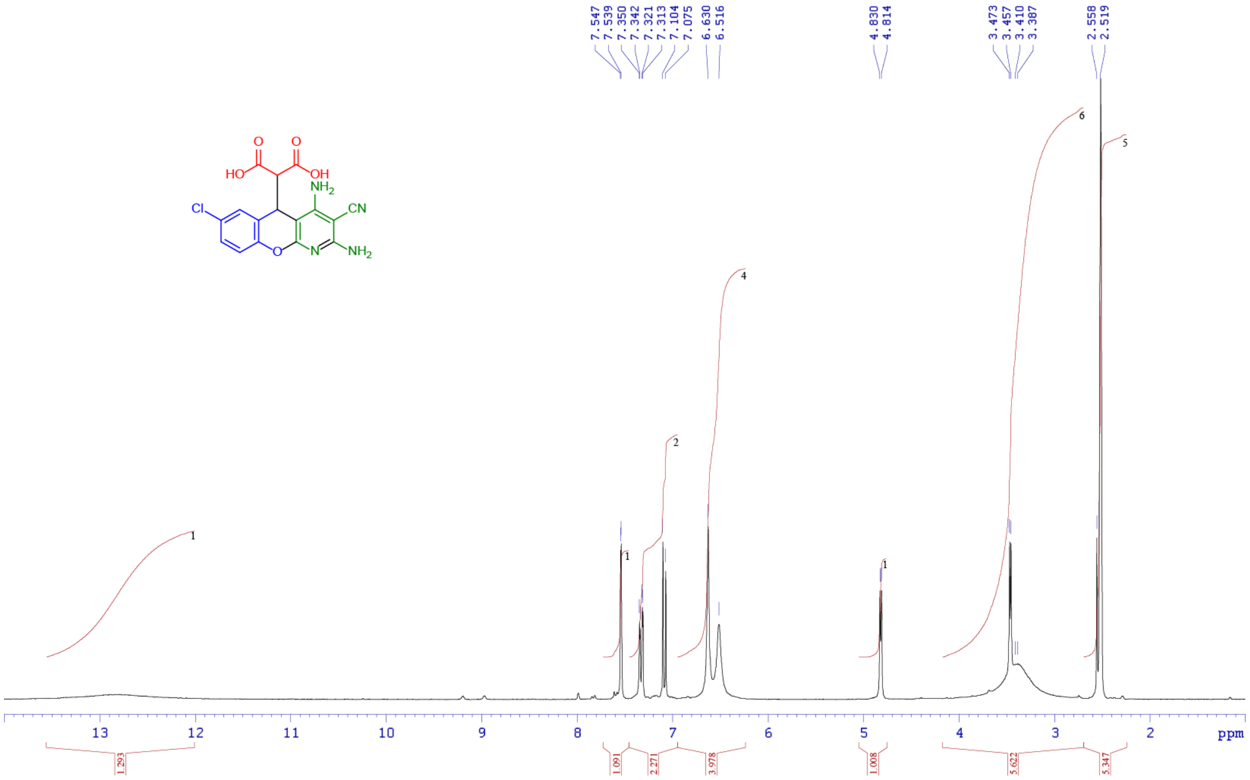

Figure S10. ^{13}C NMR spectrum of 2-(2,4-diamino-7-chloro-3-cyano-5*H*-chromeno[2,3-*b*]pyridin-5-yl)malonic acid **4e** in DMSO-d_6.

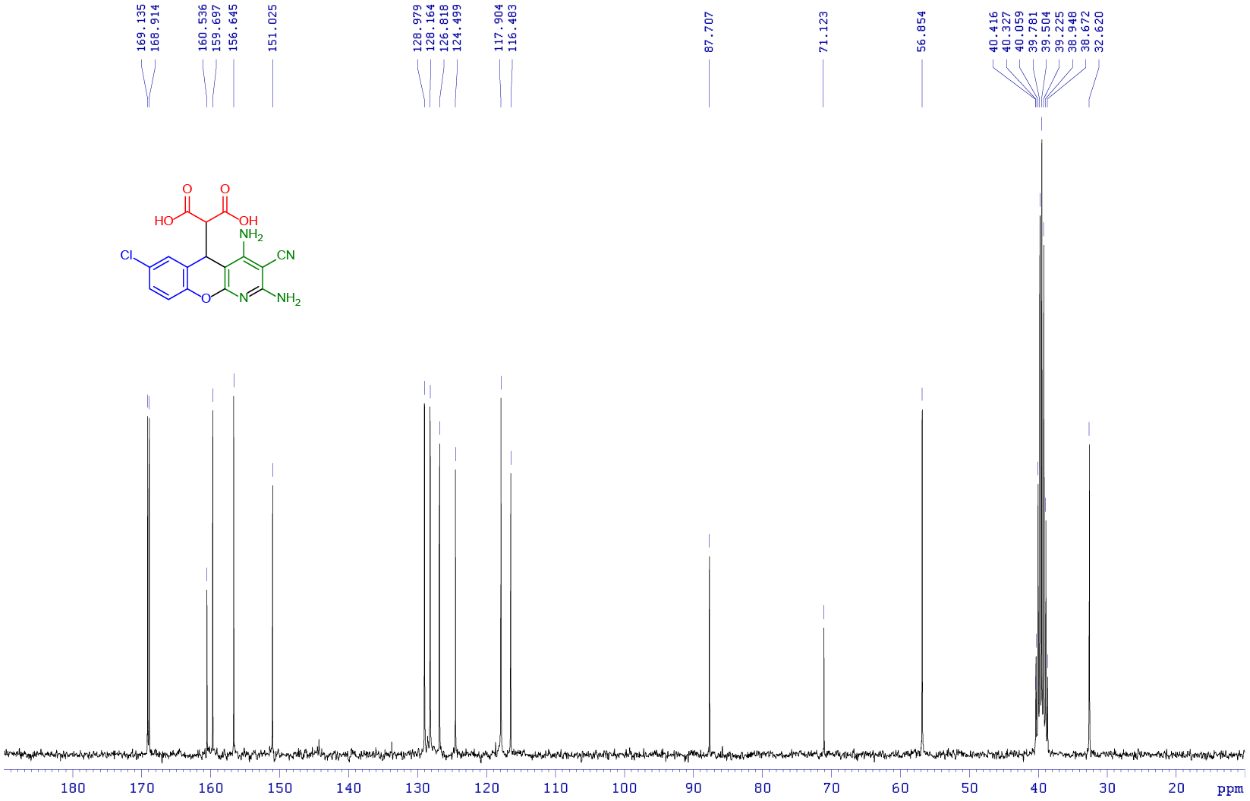

Figure S11. ^{1}H NMR spectrum of 2-(2,4-diamino-7-bromo-3-cyano-5*H*-chromeno[2,3-*b*]-pyridin-5-yl)malonic acid **4f** in DMSO-d_6.

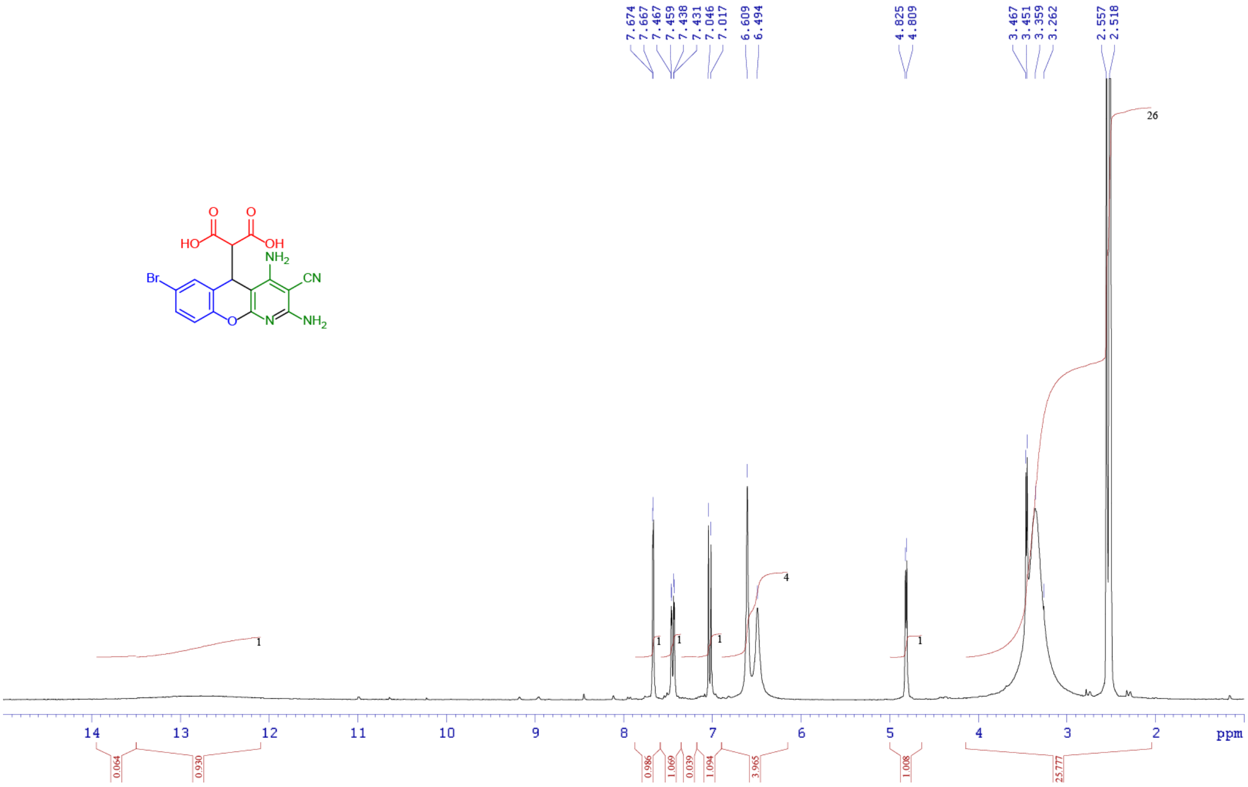

Figure S12. ^{13}C NMR spectrum of 2-(2,4-diamino-7-bromo-3-cyano-5*H*-chromeno[2,3-*b*]-pyridin-5-yl)malonic acid **4f** in DMSO-d_6.

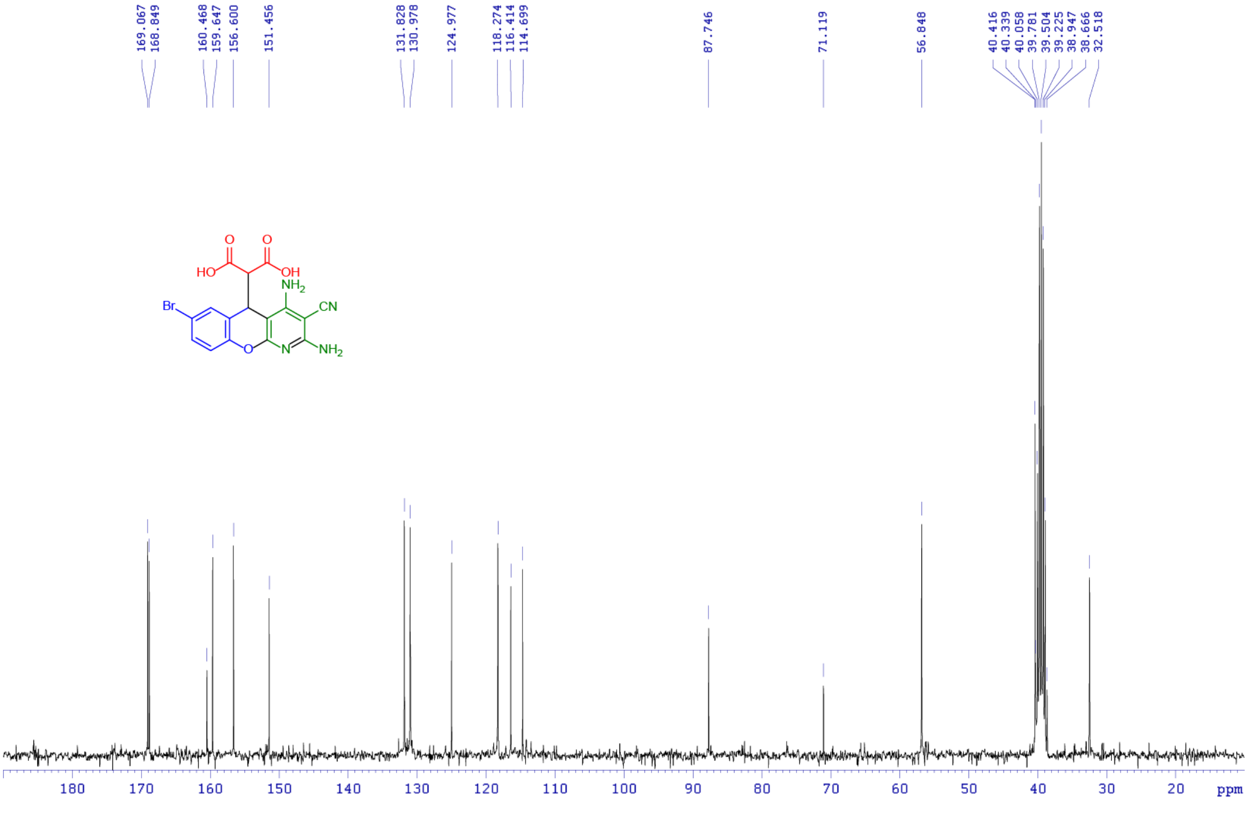

Figure S13. ^{1}H NMR spectrum of 2-(2,4-diamino-7-bromo-3-cyano-9-methoxy-5*H*-chromeno[2,3-*b*]pyridin-5-yl)malonic acid **4g** in DMSO-d_6.

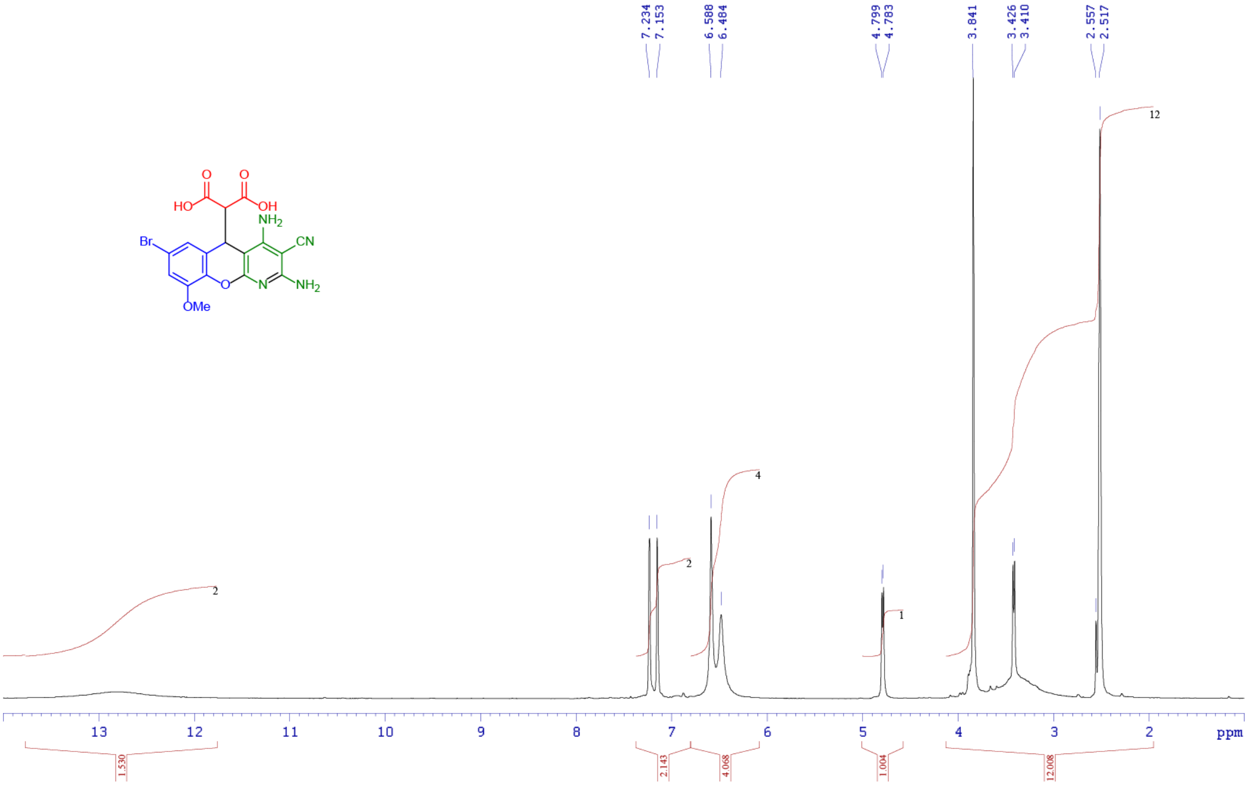

Figure S14. ^{13}C NMR spectrum of 2-(2,4-diamino-7-bromo-3-cyano-9-methoxy-5*H*-chromeno[2,3-*b*]pyridin-5-yl)malonic acid **4g** in DMSO-d_6.

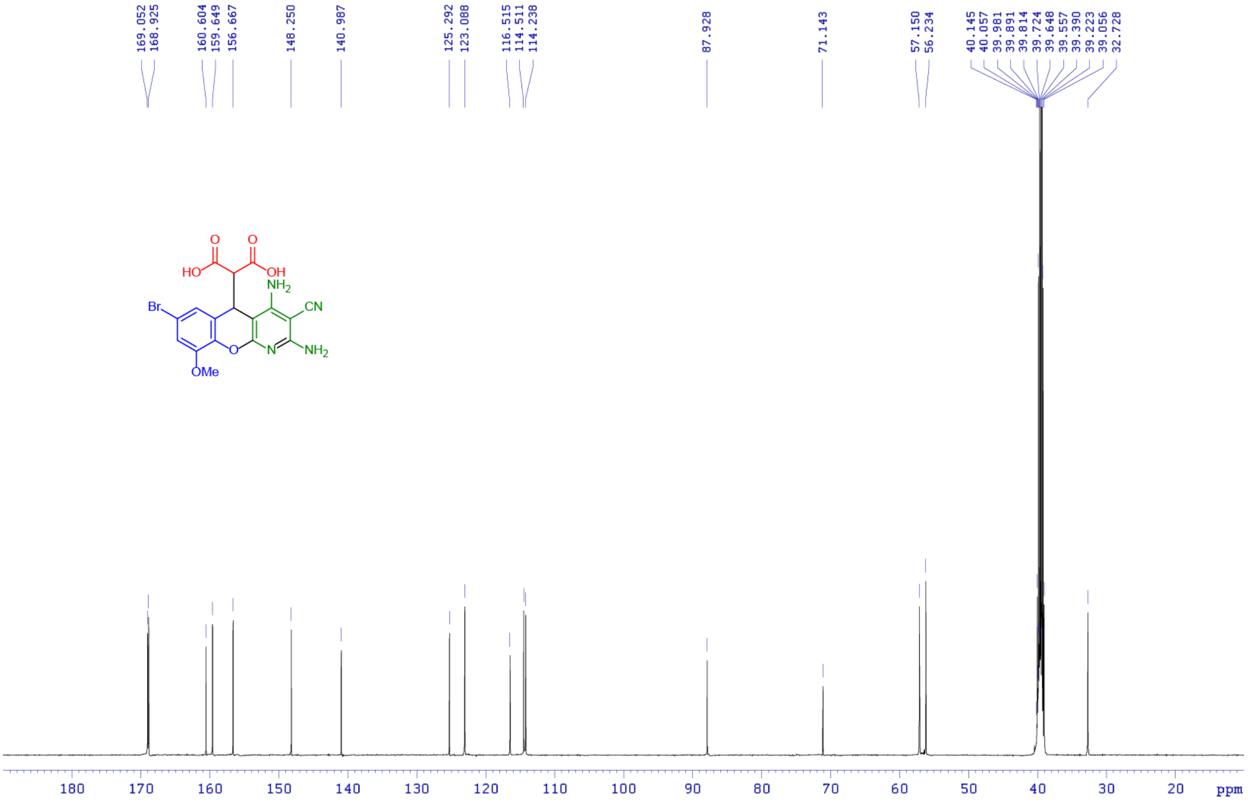

Figure S15. ^{1}H NMR spectrum of 2-(9,11-diamino-10-cyano-12*H*-benzo[5,6]chromeno[2,3-*b*]-pyridin-12-yl)malonic acid **4h** in DMSO-d_6.

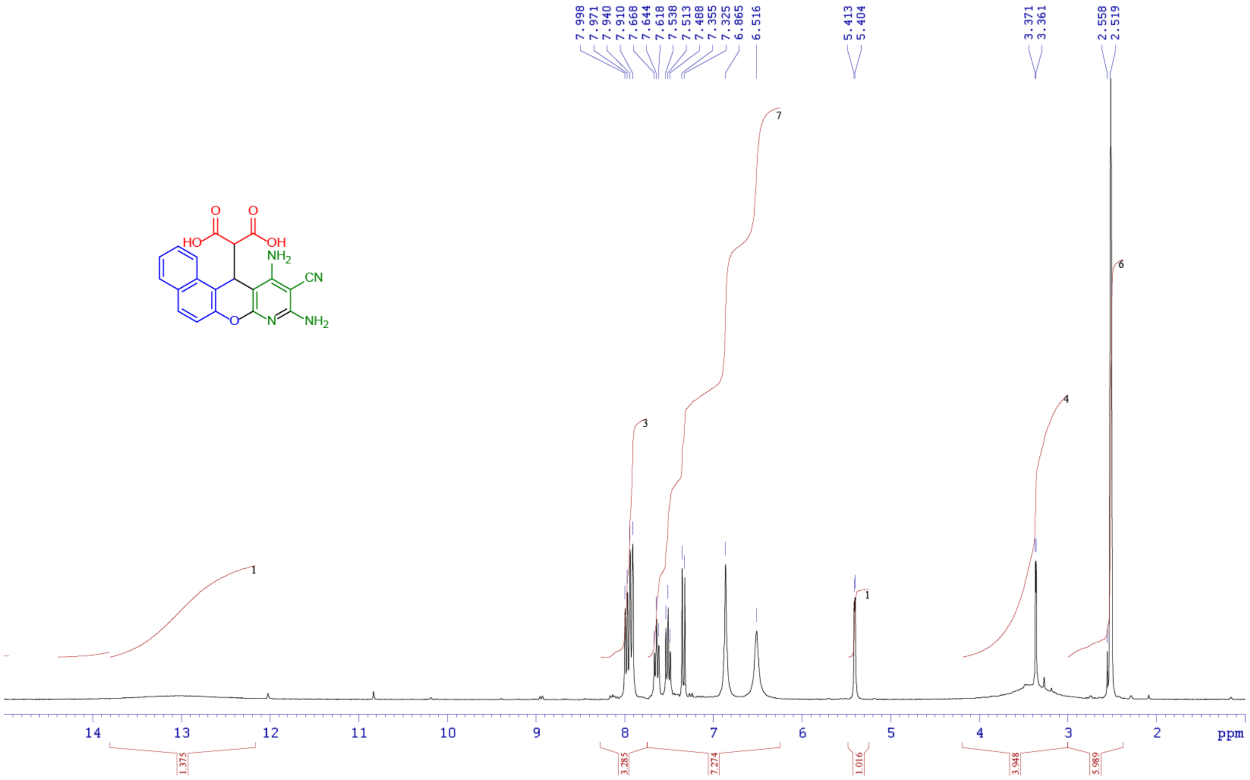

Figure S16. ^{13}C NMR spectrum of 2-(9,11-diamino-10-cyano-12*H*-benzo[5,6]chromeno[2,3-*b*]-pyridin-12-yl)malonic acid **4h** in DMSO-d_6.

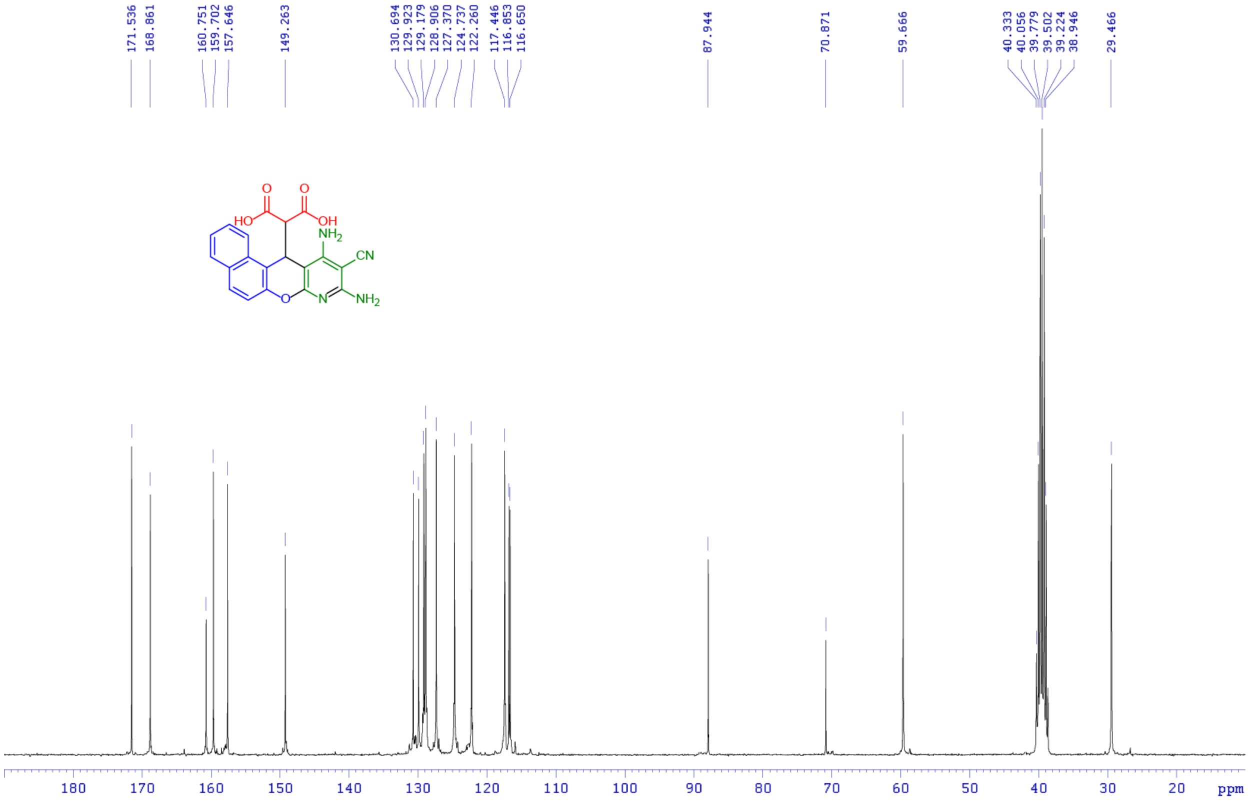

2. 2D NMR data for compound 4f

Figure S17. ^{1}H-^{13}C HMBC NMR spectrum of 2-(2,4-diamino-7-bromo-3-cyano-5*H*-chromeno[2,3-*b*]-pyridin-5-yl)malonic acid **4f** in DMSO-*d*$_6$.

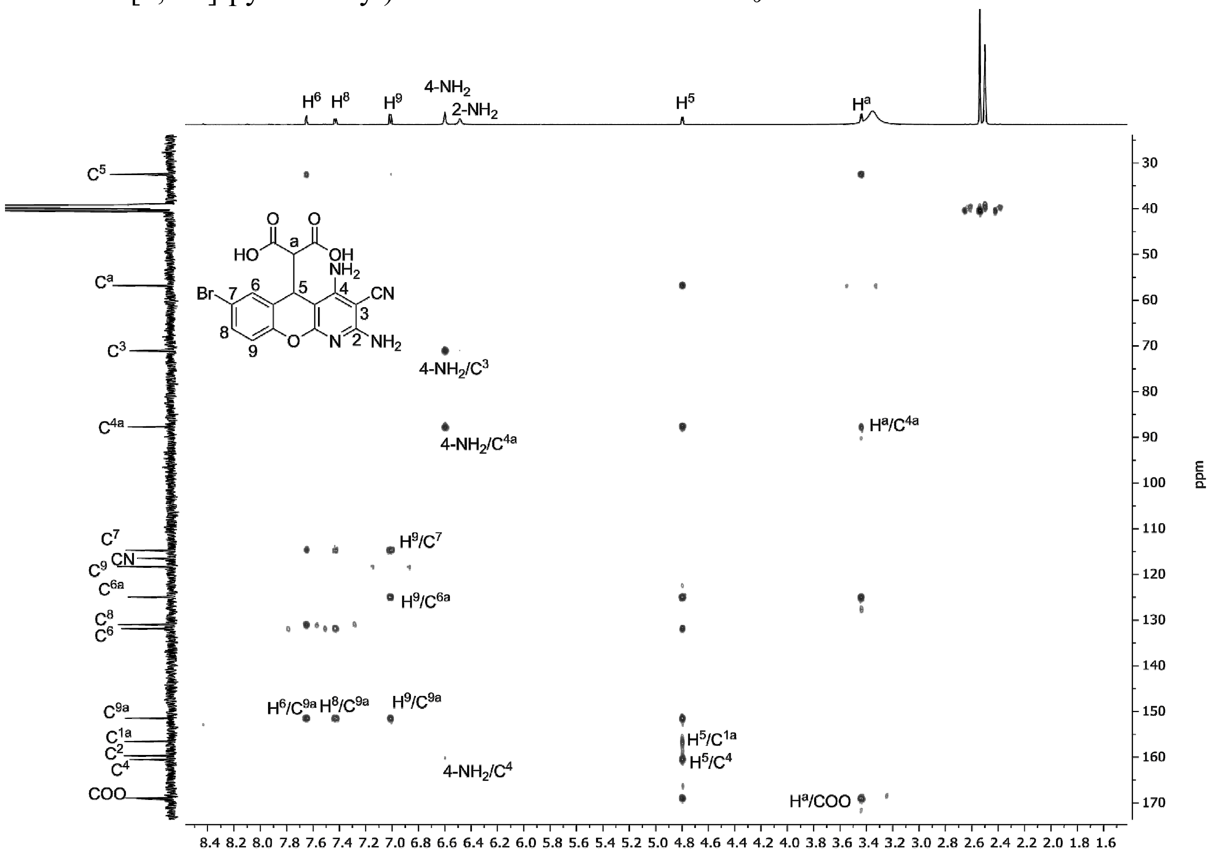

Figure S18. ^{1}H-^{13}C HSQC NMR spectrum of 2-(2,4-diamino-7-bromo-3-cyano-5*H*-chromeno[2,3-*b*]-pyridin-5-yl)malonic acid **4f** in DMSO-*d*$_6$.

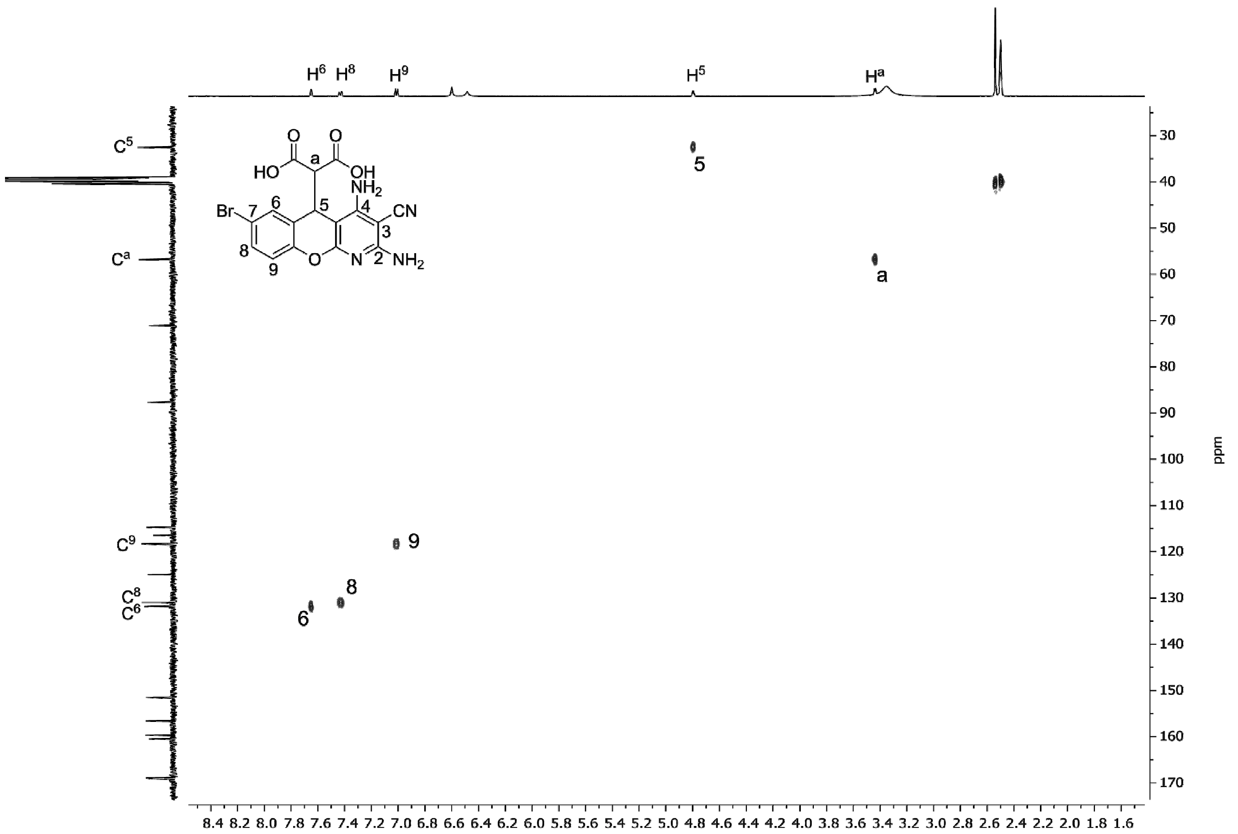

3. ^{1}H NMR monitoring spectra (300 MHz, 313 K)

Figure S19. ^{1}H NMR spectrum of reaction spectrum of salicylaldehyde **1a**, malononitrile dimer **2** and malonic acid **3** in DMSO-d_6 (immediately after mixing the starting materials).

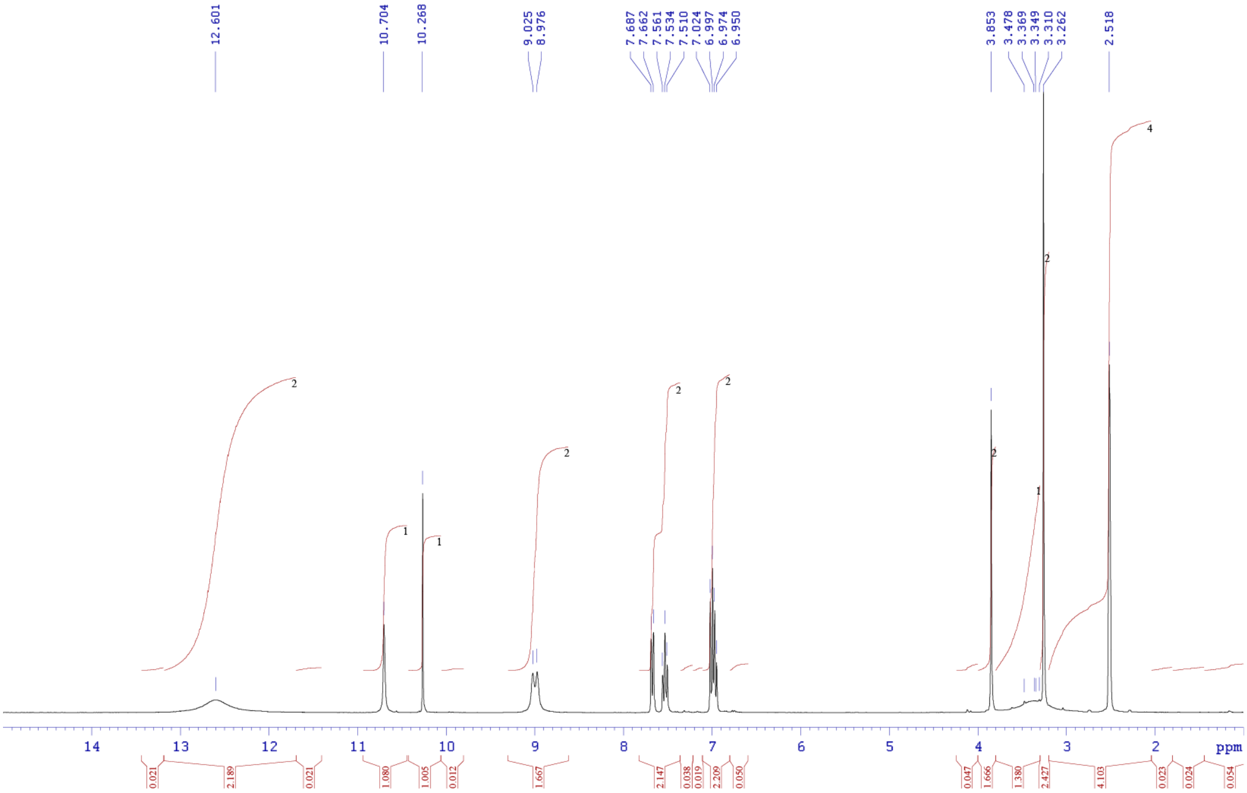

Figure S20. ^{1}H NMR spectrum of reaction spectrum of salicylaldehyde **1a**, malononitrile dimer **2** and malonic acid **3** in DMSO-d_6 (15 min after the start of heating).

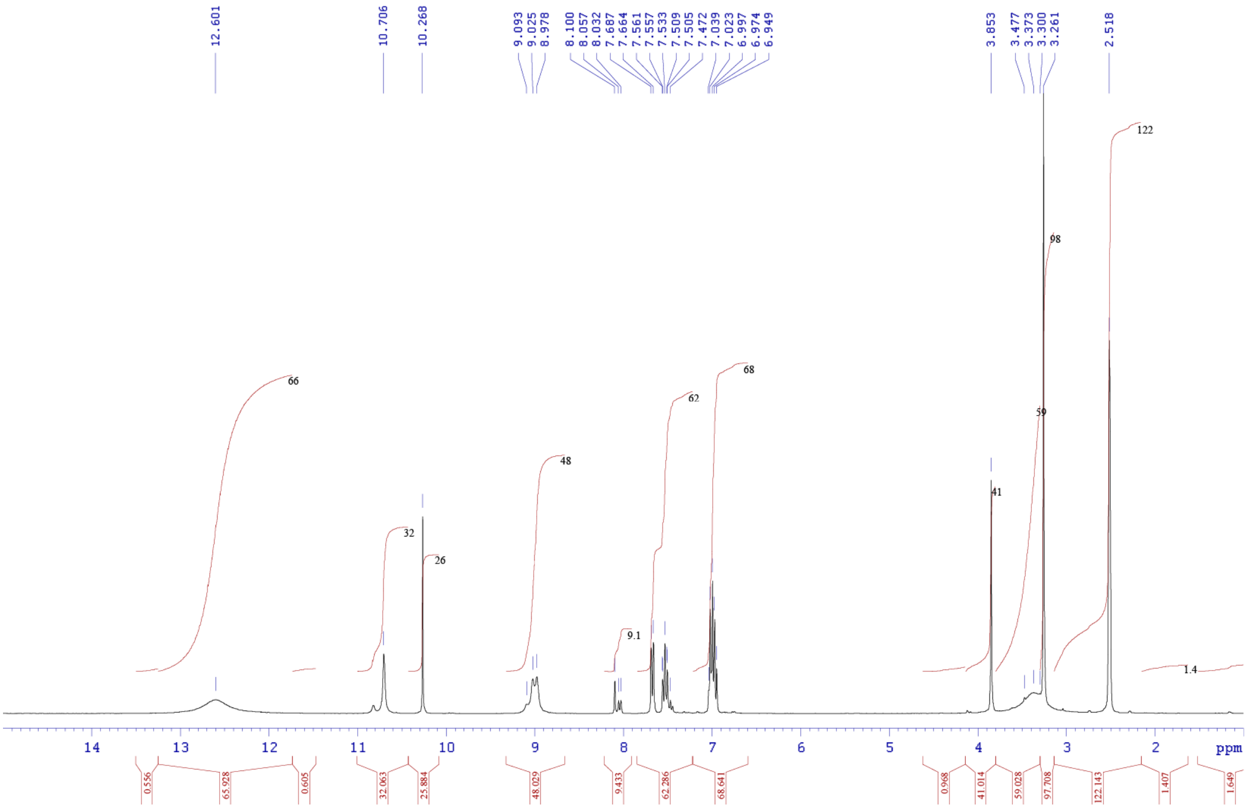

Figure S21. ^{1}H NMR spectrum of reaction spectrum of salicylaldehyde **1a**, malononitrile dimer **2** and malonic acid **3** in DMSO-d_6 (45 min after the start of heating).

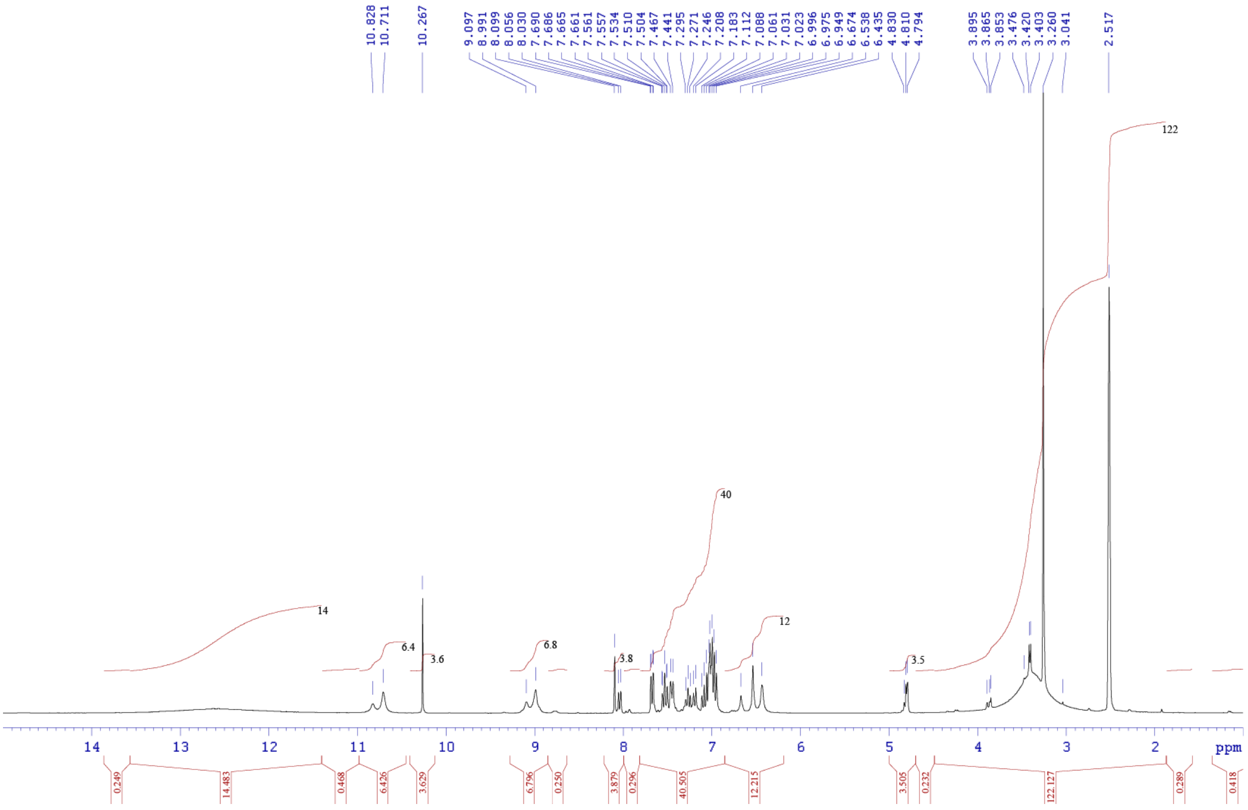

Figure S22. ^{1}H NMR spectrum of reaction spectrum of salicylaldehyde **1a**, malononitrile dimer **2** and malonic acid **3** in DMSO-d_6 (1 h 15 min after the start of heating).

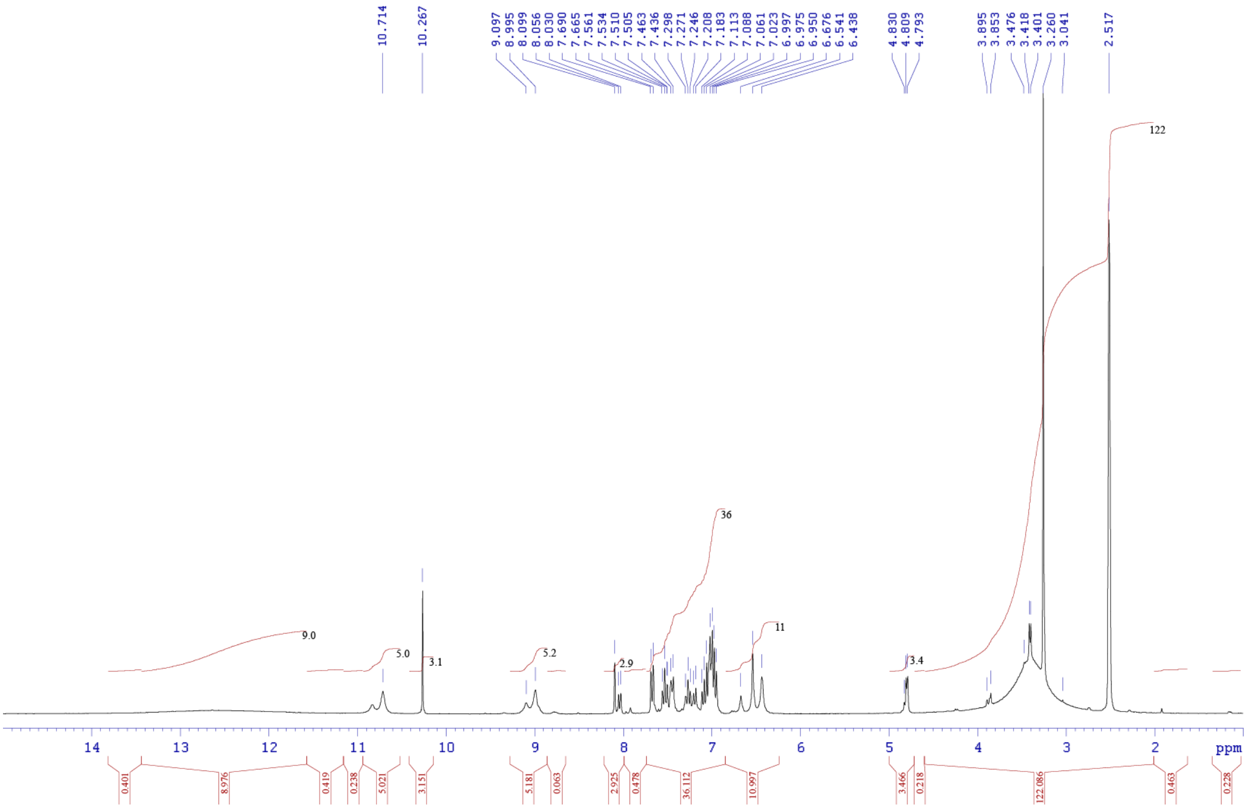

Figure S23. ^{1}H NMR spectrum of reaction spectrum of salicylaldehyde **1a**, malononitrile dimer **2** and malonic acid **3** in DMSO-d_6 (2 h after the start of heating).

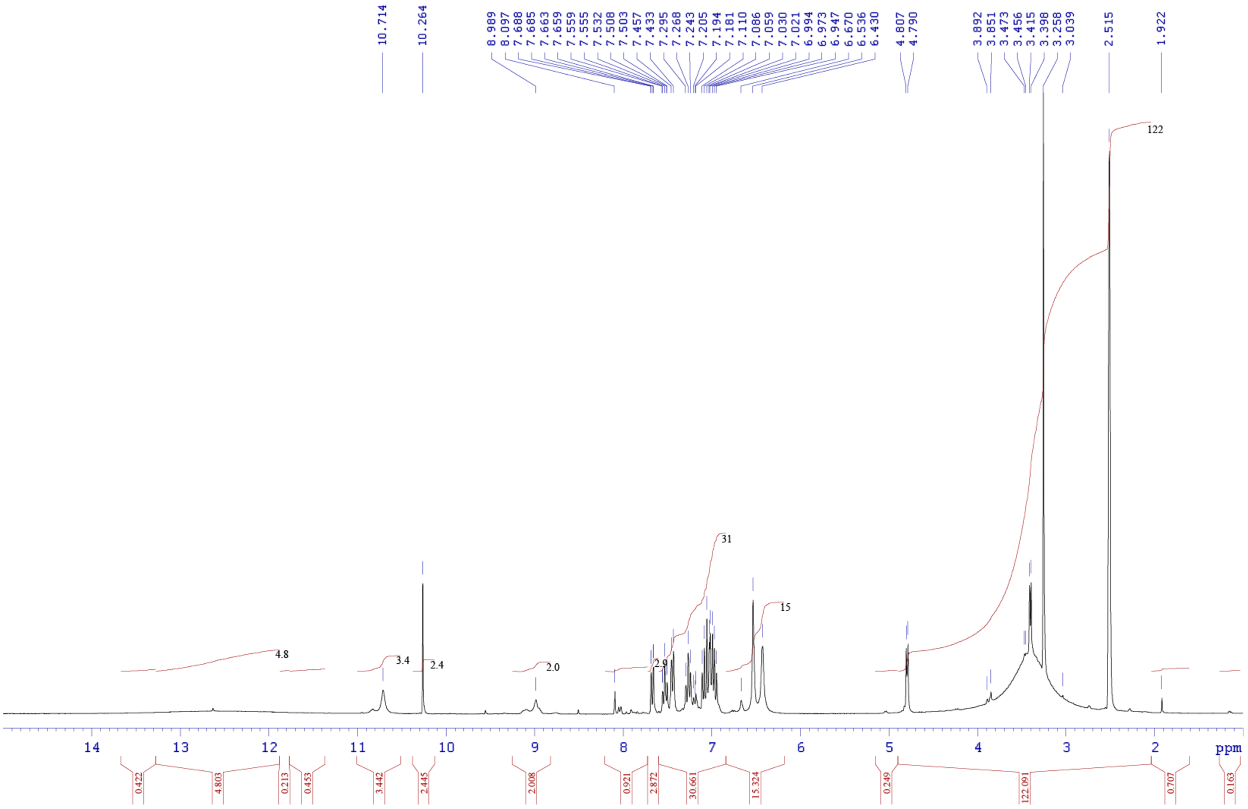

Supplement: Supplementary file 1 [file molecules-26-06839-s001.zip › molecules-1466343-supplementary.pdf]
